# Prophylactic and therapeutic efficacy of *Acinetobacter* phage RM_A1 against carbapenem-resistant *Acinetobacter baumannii* with no cytotoxicity to human skin cells

**DOI:** 10.1186/s12866-025-04698-7

**Published:** 2026-02-03

**Authors:** Rabab M. Soliman, Ahmed B. Barakat, Ayman El-Shibiny, Iman Mohamed Amin Elkholy, Ahmed Askora, Azza G. Kamel, Hagar A. Elshibiny, Marwa M. Gado

**Affiliations:** 1 Department of Microbiology, Faculty of Science, Ain Shams University, El- Khalyfa El-Mamoun Street, Abbasya, Cairo Egypt; 2 Center for Microbiology and Phage Therapy, Zewail City of Science and Technology, Giza, 12578 Egypt; 3 Faculty of Environmental Agricultural Sciences, Arish University, Arish, Egypt; 4 Ain Shams Specialized Hospital, Ain Shams University, El-Khalyfa El-Mamoun Street, Abbasya, Cairo Egypt; 5 Department of Microbiology and Botany, Faculty of Science, Zagazig University, Zagazig, 44519 Egypt; 6 Center for Genomics, Zewail City of Science and Technology, Giza, 12578 Egypt

**Keywords:** Prophylactic effect, Therapeutic effect, Carbapenem-resistant *A. baumannii*, Wound infection, RM_A1 phage, Biofilm formation, Cytotoxicity

## Abstract

**Background:**

The rise in multidrug-resistant (MDR) bacteria has rendered common first- and last-line antibiotics ineffective, posing a serious threat to human health. Additionally, the global rise of wound infections caused by carbapenem-resistant *Acinetobacter baumannii* indicates that the “post-antibiotic” era has begun. Consequently, alternative antimicrobial therapies are urgently required, and the reuse of phages is a promising choice.

**Methods:**

Twenty-six strains of *A. baumannii* were isolated from wound-infected patients, identified by the Vitek 2 automated system, and confirmed by 16 S rDNA sequencing. The strains’ susceptibility to antibiotics and their ability to develop biofilms were then studied. *Acinetobacter* phage RM_A1 was isolated and characterized, and its lytic activity against *A. baumannii* was tested in vitro via time-killing curve and antibiofilm formation assays. The phage’s stability was also tested under various conditions. Genomic analysis was performed to characterize the phage and its virulence. Finally, the phage was assessed as a therapeutic and prophylactic agent against carbapenem-resistant *A. baumannii in vitro* by using human skin cells.

**Results:**

The clinical strains of *A. baumannii* were found to be resistant to the carbapenem antibiotics, exhibiting high MAR index values and a strong ability to form biofilm. The isolated RM-A1 phage had a myoviral morphology and a 43,994 bp double-stranded DNA genome encoding 84 open reading frames (ORFs), with no genes linked to antibiotic resistance or pathogenicity. This phage possesses a broad host range, with a lysis spectrum of 85% against clinical isolates. It also possesses a large burst size and a short adsorption rate. It was capable of withstanding temperatures as high as 90 °C, was pH-stable, and remained viable for 45 min when exposed to UV light. There was no significant drop in the vitality of HSF cells observed following phage treatment, proving that the phage is safe for use. In vitro results showed that the phage was able to inhibit and eliminate biofilm formation and significantly reduce bacterial growth.

**Conclusion:**

Our research explores the potential use of RM_A1 phage as an alternative therapeutic agent for combating carbapenem-resistant *A. baumannii*.

**Supplementary Information:**

The online version contains supplementary material available at 10.1186/s12866-025-04698-7.

## Background

The skin is the largest and outermost organ covering the entire body, and skin wounds are among the most prevalent physical injuries in human history [1]. As soon as skin injury occurs, the wound-healing process is initiated to restore the skin’s structure and functionality [2]. Infection is a significant barrier to wound healing and is one of the most common nosocomial diseases, resulting in at least 10,000 fatalities for every million patients [3]. *Acinetobacter baumannii* (*A. baumannii*) is one of the most common causes of wound infections; it colonizes the wound site and then spreads via the bloodstream into internal organs, resulting in skin necrosis and other severe consequences that can be fatal [4, 5].

Consequently, *A. baumannii* is at the top of the World Health Organization’s (WHO) list of critical pathogens that require immediate access to new antibiotics [6]. The Centers for Disease Control and Prevention of America (CDC), the Infectious Diseases Society of America (IDSA), and the European Centre for Disease Prevention and Control (ECDC) have considered it a critical issue [7]. Furthermore, according to the WHO [8], it is also one of the most threatening ESKAPEE organisms, which include *Enterococcus faecium*,* Staphylococcus aureus*,* Klebsiella pneumoniae*,* A. baumannii*,* Pseudomonas aeruginosa*,* Enterobacter species*, and *E. coli*.

Numerous factors, such as antibiotic inactivation enzymes, target site modifications, overexpression of efflux pumps, and porin loss, contribute to *A. baumannii’s* antibiotic resistance [9]. One of *A. baumannii’s* main resistance strategies is the formation of biofilms. *A. baumannii* biofilms are essential for shielding bacteria from host immunological reactions and can withstand antibiotics up to 1000 times better than planktonic cells [10]. The development of biofilms by *A. baumannii* is essential for both pathogenicity and antibiotic resistance, and it is also thought to play a significant role in delaying the healing of wounds and causing chronic infections [11].

According to worldwide studies, over 45% of all *A. baumannii* isolates have been reported to be multidrug-resistant, with the percentage rising to 90% in the Middle East, Turkey, and Greece. In Egypt, between 30% and 100% of *A. baumannii* isolates are classified as multidrug-resistant (MDR), and between 26.6% and 100% of isolates have been determined to be carbapenem resistant [12].

Statistics indicated that approximately one million people worldwide die from antibiotic-resistant diseases each year, and if alternative therapies are not available, this number is expected to rise to 10 million annually by 2050 [13]. The potential of current drugs to inhibit MDR isolates has become limited or nonexistent, indicating the end of the antibiotic era and the beginning of a post-antibiotic era [14]. Therefore, the clinical and economical impacts of MDR bacteria have led to the need for alternate antibacterial agents [15]. Bacteriophages (phages) are a possible option; they were utilized as therapeutic agents for controlling pathogenic bacteria twenty years before the first antibiotics were used in hospitals. However, the appearance of broad-spectrum antibiotics in the 1940s rapidly displaced and stopped the development of phage therapies in most parts of the world [16, 17].

Although they are harmless to humans, bacteriophages are viruses that can infect and multiply inside specific bacterial cells. On Earth, they are the most prevalent biological entities [18], with an estimated global population of 10^31^ [19]. Phages are approximately one-fortieth the size of bacteria, and most of them have a hollow tube tail that allows their DNA to be injected into host cells and a protein head that contains the genetic material (DNA). Phages have special features that make them effective therapeutic agents, offering a possible solution to the MDR challenge. They are both self-limiting and self-replicating, continuing to grow and spread to deeper infection. In contrast, antibiotics decrease their concentrations at the site of infection. Unlike antibiotics, which are active against a group of bacteria, phages are lytic against particular bacteria, allowing for more accurate targeting [20].

Phage therapy’s effectiveness has been assessed against a number of MDR bacteria, including *A. baumannii*, which is extremely challenging to treat with conventional antibiotics. Many studies have demonstrated that phage therapy works efficiently against carbapenem-resistant *A. baumannii* [21]. The effectiveness of polymyxin B and two bacteriophages in treating carbapenem-resistant *A. baumannii* was compared by Zhou et al. [22], in which the study used the *Galleria mellonella* larva model, and the results showed that phage therapy increased the survival of *A. baumannii*-infected larvae by up to 75%, whereas polymyxin B only very slightly raised the survival rate of *A. baumannii*-infected larvae. A different study evaluated bacteriophages’ ability to destroy colistin-resistant *A. baumannii*. After just 40 min of a single phage treatment, the study found that the number of colistin-resistant *A. baumannii* had significantly decreased [23]. Additionally, phage therapy proved successful effect against strains of XDR *A. baumannii*. Wang et al. [24]. evaluated the φkm18p phage’s ability to eliminate XDR *A. baumannii*. According to the study, mice infected with XDR *A. baumannii* showed a significant improvement in survival of up to nearly 100% when treated with monophage.

Human cell lines are frequently employed as models for drug screening and toxicity investigations, as well as for predicting clinical reactions to medications. They also demonstrated how bacteria adhere to and proliferate in the presence of human cells [25]. Several studies have evaluated specific features of phage therapy using cell lines. Alemayehu et al. [26] showed that phages successfully destroyed *P. aeruginosa* growing on a cystic fibrosis bronchial epithelial cell line. In another analysis, the safety of *A. baumannii* phage therapy was assessed using a lung epithelial cell [27]. All previous studies support the efficacy of using therapeutic phages against multi drug-resistant *A. baumannii* infections without harming the cell lines that were employed.

In this work, we investigated *Acinetobacter* phage RM_A1’s prophylactic and therapeutic effectiveness against carbapenem-resistant *A. baumannii in vitro.*

## Materials and methods

### Isolation and identification of bacteria

Twenty-six pathogenic *A. baumannii* were isolated from wound infections from Abou Elazayem hospital in Cairo, Egypt, over thirteen months, from July 2023 to August 2024. The first step involved inoculating swabs from wound infections on MacConkey and blood agar medium, followed by the Gram stain, and then further identification was revealed by using the VITEK-2 system (bioMérieux, Marcy l’Etoile, France) [28]. The collection process was conducted in accordance with the Declaration of Helsinki and was approved by the Research Ethics Committee at Ain Shams University (Approval No. ASU-SCI/MICR/2023/9/7).

### Antibiotic sensitivity test

Two milliliters of each *A. baumannii* strain suspension were prepared for the susceptibility test, and they were automatically supplied into the VITEK-2 AST system (bioMérieux, Marcy l’Etoile, France) using the GNB susceptibility testing cards and the 2.01 version software. The 17 tested antibiotics were from 8 different classes: tetracycline (tigecycline 16 µg/ml), polymyxin (colistin 0.5 µg/ml), beta-lactam combination (ampicillin-sulbactam 32 µg/ml, piperacillin 128 µg/ml), carbapenem (imipenem 16 µg/ml, meropenem 16 µg/ml, ertapenem 16 µg/ml), cephalosporin (cefepime 64 µg/ml, ceftriaxone 64 µg/ml, ceftazidime 64 µg/ml, ceftazidime-avibactam 64 µg/ml, and cefazolin 64 µg/ml), fluoroquinolones (levofloxacin 8 µg/ml, ciprofloxacin 4 µg/ml ), aminoglycosides (gentamycin 16 µg/ml, tobramycin 16 µg/ml), and sulphonamides (trimethoprim-sulfamethoxazole 160 µg/ml ) [29]. Data of antibiotic susceptibility were analyzed using the CLSI recommendations, which divide antibiotic susceptibility into three categories: sensitive, intermediate, and resistant. The MAR index is calculated by dividing the number of antimicrobial agents to which a bacterial isolate is not susceptible (either resistant or intermediately susceptible) by the total number of antimicrobial agents that were tested [30].

### Detection of biofilm formation by microtiter plate assay

The microtiter plate (MtP) assay is a quantitative technique to measure biofilm formation using a microplate reader. A bacterial suspension is prepared in TSB with 1% glucose added, and 200 µl of the suspension is then inoculated, with 10^6^ cfu/ml, in a 96-well, flat-bottomed, sterile polystyrene microplate. The microplates are incubated at 37 °C for 24 h [31, 32]. After disposing of the planktonic cells, the wells were washed twice with distilled water. The plates were then allowed to air dry to make direct fixation of the biofilm easier. After staining with 0.1% crystal violet (CV) solution (Solarbio, Beijing, China), 30% acetic acid was used for destaining. A microplate reader was used to measure each well’s absorbance at 595 nm. (BioTek, Synergy, USA) [33]. Blanks are inoculated wells that contain sterile TSB supplemented with 1% glucose and are used as negative controls. To determine whether or not isolates are forming biofilms, the blank absorbance values are analyzed. Biofilms are produced by isolates whose OD values are higher than those of the blank well. Based on optical density (OD) measurements, biofilm production can be divided into four types. Non-producer is indicated by ODs ≤ ODc, weak producer by ODc ≤ ODs ≤ 2 ×ODc, moderate producer by 2 ×ODc ≤ ODs ≤ 4 ×ODc, and strong producer by ODs > 4 ×ODc. ODs stand for the OD of the experimental samples, while ODc is the OD of the negative control [34]. This experiment was conducted in triplicate.

### Molecular identification of bacterial isolate

*A. baumannii* strain A 18 molecular identification involved the amplification and sequencing of the 16S rRNA gene using primers (F) 5’-GGGGGATCTTCGGACCTCA-3’ and (R) 5’-TCCTTAGAGTGCCCACCCG-3 [35]. The sequencing was carried out using the BigDye Terminator Cycle Sequencing Kit (V. 3.1, Applied Biosystems) according to the manufacturer’s protocol, and it was examined in an Applied Biosystems analyzer. Finally, the 16 S-rDNA sequence for the strain A 18 was uploaded to the NCBI GenBank database and given an accession number [36].

### Phage isolation, purification, and propagation

A number of sewage water samples from Aboalazium Hospital, Cairo, Egypt, were collected to isolate phages. The enrichment process is performed according to Adam [37]. Briefly, 9 mL of each sewage sample were mixed with 1 mL of an of *A. baumannii*, incubated for 24 h at 37 °C, and then centrifuged for 20 min at 5000 rpm, and the supernatant was filtered using a 0.22 μm syringe filter. For a spot test, 100 µl of exponential-phase bacterial host culture was combined with 4 mL of soft agar (0.7% w/v agar). The mixture was then used at a suitable temperature between 45 and 50 °C and was put into a tryptic soya agar (TSA) plate. Ten microliters of the supernatant test sample were spotted on the bacterial layer. The plates were incubated for 24 h at 37 °C. After incubation, the results determined if any clear zones were observed on the plates or not. A single plaque was then selected using a sterile pipette tip, suspended in 100 µl of Gelatin-SM buffer [5.8 g NaCl, 2.0 g MgSO_4_-7H_2_O, 50 mL 1 M Tris-HCl pH 7.4 in 1-liter dH_2_O], and refrigerated at 4 °C for 4 h. The plaque assay experiment was then carried out by tenfold serial dilution. To obtain a single pure phage, the plaque assay was then carried out many times [38]. In order to propagate the quantity of purified phages, 100 µl of a single phage was added to 10 mL of the bacterial host culture. The concentration of these purified phages was then determined using the plaque assay [39]. Three parallel groups were used for each experiment.

### Pulsed‑Field Gel Electrophoresis (PFGE)

The genome size was determined, and the isolated phage’s purity was confirmed using PFGE analysis [40, 41] with some modifications. Briefly, 100 µl of 1.4% plug agarose was combined with 100 µl of phage suspension (10^10^ PFU/mL). After solidifying, the plugs were then submerged in lysis buffer (1 mg/mL Proteinase K [ThermoFischer Scientific, USA]), 0.2% w/v SDS [Sigma Aldrich, Gillingham, UK], 100 mM EDTA, and 1% w/v N-Lauryl sarcosine [Sigma Aldrich, Gillingham, UK]. Following incubation at 55 °C for 18 h, the plugs were cleaned and put on the 1.5% PFGE agarose gel using the Lambda PFG Ladder (Biolabs, UK). The Bio-Rad CHEF DRII system (Bio-rad, USA) was used to conduct the PFGE for 18 h at 200 V (6 V/cm) with a switch time of 30 to 60 s. This experiment was conducted in triplicate.

### Host range analysis

The spot test was used to examine the isolated phage’s inhibition activity against 26 clinical isolates of *A. baumannii*. Bacterial strains’ vulnerability to the phage has been demonstrated by the development of clear zones, or bacterial lysis [42]. This experiment was conducted in triplicate.

### Efficiency Of Plating (EOP)

A further evaluation was conducted to determine the effectiveness of phages against susceptible strains of *A. baumannii*. For this experiment, the phage titer is serially diluted 10-fold (10^1^ to 10^8^). Each susceptible bacterial isolate was observed in triplicate on a new lawn using ten microliters of each dilution. To determine the EOP, the PFUs produced by the phage on each bacterial host were counted after an overnight incubation period. The average was then divided by the highest recorded PFU count [43]. The EOP value was divided into four groups and categorized as “high production” when the ratio was 0.5 or higher, “medium production” efficiency was defined as an EOP of 0.1 or higher but less than 0.5, and “low production” efficiency as being between 0.001 and 0.1. An inefficient EOP was defined as one that was equal to or less than 0.001 [44]. This experiment was conducted in triplicate.

### Optimal Multiplicity of Infection (MOI) determination

The MOI was carried out according to Liu et al. [45]. An *A. baumannii* phage was incubated with the *A. baumannii* culture (10^6^ CFU/mL) in different MOIs (100, 10, 1, 0.1, 0.01, and 0.001) for five hours at 37 °C and 170 rpm. The plaque assay method was used to determine the phage titers at various MOI. Three parallel groups were used for this experiment.

### Phage adsorption rate

According to Shaykh-Baygloo and Bouzari [46], with some modifications, the phage suspension was mixed with an *A. baumannii* culture at an MOI of 0.1 and incubated at 37 °C to measure the adsorption rate. After 3, 5, 8, 10, 12, 15, and 20 min, samples (1 ml) were collected, centrifuged at 8000 rpm for 1 min at 4 °C, and the supernatants were titrated using the plaque assay method to determine the amounts of unadsorbed phages. The entire procedure has been carried out three times.

### One-step growth curve

A one-step growth experiment was conducted to calculate the phage’s latent period, which is the time between the phage’s adsorption to the bacterial cell and the release of phage progeny, as well as its burst size, which is the quantity of phage particles released following infection of a single host cell and completion of one replication cycle. One milliliter of the phage suspension was added to nine milliliters of *A. baumannii* culture at an MOI of 0.1, and the mixture was left to adsorb for ten minutes at 37 °C. To get rid of the unadsorbed phages, the mixture was centrifuged for four minutes at 10,000 rpm. Ten milliliters of TSB (37 °C) were used to resuspend the infected bacterial cells, which were then incubated at 37 °C. Samples were collected at 5-min intervals up to 40 min and 10-min intervals up to 60 min and immediately titrated [47]; the burst size was determined by dividing the titer of released virions at plateau by the titer of the initial virions [48]. This assay was conducted in triplicate.

### Electron microscopy examination

After placing one drop of the isolated phage suspension (10^10^ pfu/ml) on a copper grid coated with 200 mesh carbon and letting it absorb for around 20 min, the extra liquid was wiped off using filter paper. A JOEL-JEM-1010 electron microscope running at 80 KV was used to examine the grids after they had been negatively stained for 90 s with 2% uranyl acetate (pH 4.5) and allowed to dry. (Electron microscope unit, Regional Center for Mycology and Biotechnology, Al-Azhar Univ., Cairo) [49].

### Phage physicochemical stability

The phage stability was conducted under various conditions of temperature, pH, and UV. The thermal stability of *A. baumannii* phage (at a concentration of 10^10^ PFU/ml) was measured over a period of one hour, using a water bath. The tested temperatures included 4 °C, 25 °C, 37 °C, 50 °C, 60 °C, 70 °C, 80 °C, 90 °C and 100 °C. Following with the serial dilutions of *A. baumannii* phage, the standard double layer technique were applied and after incubation, the phage’s titers were determined. The assessment of the phage’s UV stability was conducted at different time points of 15, 30, 45, and 60 min. Ten ml of phage lysate in a petri dish without a cover is exposed to the UV lamp of the biological safety cabinet with an intensity of around 253.7 nm. The standard double-layer technique was used to enumerate phage titers. pH stability of the phage was determined through incubation for 1 h at different pH values from 1 to 14, then The double-layer technique was performed to determine phage titers [48, 50]. Three parallel groups were used for each experiment.

### Time‑killing curve

A time-killing curve assay was carried out to examine the antibacterial effect of phage RM_A1 against the host strain. First, a day-cultured bacteria was diluted with TSB to prepare a 10^6^ CFU/mL bacterial suspension. Next, 180 µL of the bacterial suspension was distributed into 96-well plates. Then, each well was infected with phage (20 µL) at different MOIs (100, 10, 1, 0.1, 0.01, and 0.001) and incubated for eight hours at 37 °C. The absorbances at OD_600_ nm were recorded using a FLUOstar Omega microplate reader (BMG LABTECH, Ortenberg, Germany) at 2-hour intervals during the experiment [51]. The untreated bacteria were employed as a control. After 8 h of incubation, the percentage of reduction in OD_600_ was determined as follows: Percentage of reduction in OD_600_ (%) = 100 x ΔOD600 of the treated sample divided by the control’s starting OD_600_ [52] This experiment was conducted in triplicate.

### Biofilm Inhibition and clearance assay

The anti-biofilm activity of the phage was determined at against A18 strain different MOIs for two different phenotypes: (i) inhibition of biofilm formation and (ii) clearance of formed biofilm. Biofilm inhibition and mature biofilm clearanceassays were both conducted in 96-well microtiter plates. In biofilm inhibition initially, a fresh TSB-diluted bacterial culture was added to wells of microtiter plates at a concentration of 10^6^ CFU/mL with a volume of 180 µL. The phage preparations were added at different MOIs (100, 10, 1, 0.1, 0.01, and 0.001) with a volume of 20 µL. After incubating the 96-well plates at 37 °C for 24 h, the planktonic cells were discarded, and the wells were washed twice with distilled water. Subsequently, the plates were air-dried to facilitate direct fixation of the biofilm. Staining using a 0.1% crystal violet (CV) solution (Solarbio, Beijing, China) was followed by destaining using 30% acetic acid. Between these steps, wells were rinsed with distilled water and left to air dry. Finally, absorbance for each well was measured at 595 nm using a microplate reader (BioTek, Synergy, USA). In the mature biofilm clearance assay, instead of immediate phage treatment, bacterial cultures were incubated for 24 h to establish the biofilm. Subsequently, phage preparations at different MOIs were added to the untreated biofilm and incubated at 37 °C for another 24 h [53] As in previous steps, the anti-biofilm activity of the RM_A1 phage at MOI 0.1 and the meropenem antibiotic (16 µg/ml) from the carbapenem class was assessed for all strains of *A. baumannii* that were susceptible to the RM_A1 phage. 

The percentage of inhibition and clearance was calculated according to the following equation: $\mathrm{Percentage}\;\mathrm{of}\;\mathrm{inhibition}=\left[\left({\mathrm{OD}}_{\mathrm{Untreated}}-{\mathrm{OD}}_{\mathrm{Treated}}\right)/{\mathrm{OD}}_{\mathrm{Untreated}}\right]\times100$. This experiment was conducted in triplicate.

### DNA isolation and purification

According to Sambrook and Russell [54], with minor adjustments, the phenol-chloroform–isoamyl alcohol technique was used to extract the phage’s genomic DNA from 10 ml of purified high-titer (10^10^ PFU/ml) phage lysate. Initially, proteinase K and sodium dodecyl sulfate (SDS) (10% w/v) were added to the phage at a final concentration of 50 ug/ml and 0.5%, respectively, then incubated for an hour at 56 °C to lyse the phage capsid and liberate the contained DNA. To extract genomic DNA from proteins, the solution was then thoroughly mixed with a volume of phenol, chloroform, and isoamyl alcohol (25:24:1). By centrifuging the mixture at 18,000 rpm for 10 minutes, the mixture was divided into two phases: an organic phase that included proteins and was disposed of, and an aqueous phase on top from which DNA was extracted. One to ten volumes of 3 M sodium acetate (pH 5.2) and 2:1 volumes of ice-cold isopropanol were used to precipitate the extracted DNA in the aqueous phase overnight at -20 °C. After discarding the supernatant, the pellet was mixed with 90% ice-cold ethanol and put into another 1.5 ml tube. After two rounds of washing in 70% ethanol, the extracted DNA pellet was allowed to dry before mixing with 100 milliliters of nuclease-free water. The FLUOstar Omega Microplate reader (BMG LABTECH, Germany) was used to measure the DNA quality and concentration.

### Genome sequencing, assembly and annotation

To sequence the isolated DNA, conventional Illumina procedures were used. In short, using the Illumina Nextera tagmentation procedure (Illumina, Cambridge, UK), a genomic library of the phage’s extracted DNA was created. FastQC v0.11.9 was used to assess the quality of the sequence readings, and Unicycler v0.4.8 was used to assemble them. The Rapid Annotation using Subsystem Technology Toolkit (RASTtk) pipeline was then used to annotate the single constructed contig [55]. A second round of annotation was carried out following RASTtk annotation in order to either confirm assigned functions or assign functions to proteins that had not yet been assigned. For that, a number of tools were utilized, such as NCBI BLASTp, HHPred, InterPro Scan, and UniProt. The Proksee web-based tool [56] constructed a genomic map of the phage.

### Bioinformatics analysis

According to Hockenberry and Wilke [57], PHAGESCOPE (Galaxy Version 1.0) predicted the phage lifestyle by using conserved domains of integrases, excisionases, recombinases, and partitioning proteins (ParA and ParB). Additionally, the phage therapeutic efficacy was determined using PhageLeads, which examines the phage genome for virulence genes, AMR, and temperate genetic markers.

### Phylogenetic analysis

The Turner et al. [58] standards were applied for doing genome-based taxonomy. We used the ViPTree server [59] to set up a proteome-based clustering technique for family-level classification. Several dsDNA prokaryotic viruses and closely related phages were used in the analysis. To create a more detailed rectangular proteome tree, phages with the greatest VipTree tBLASTx scores (SG) were chosen. VIRIDIC (Virus Intergenomic Distance Calculator) was utilized to find intergenomic similarities between the isolated phage genome and the closest phage homologs in BLASTn and other phages that were filtered from the NCBI virus database. The filtered phages’ FASTA sequence was obtained and chosen for VIRIDIC analysis. Using the conventional algorithm, VIRIDIC determines pairwise intergenomic distances and similarities in accordance with the ICTV guideline [60]. CoreGenes5 was carried out to identify shared signature genes between the phage and its close relatives [61]. Using the amino acid sequence alignments of the highly conserved signature genes that encode the DNA polymerase enzyme, the major capsid protein, and the terminase large subunit, genus- and family-level phylogeny were further confirmed. For the isolated phage and its close relatives, a conserved gene-based phylogenetic analysis was performed against an outgroup. Muscle analysis was used for the alignment of the amino acid sequences of the conserved genes of the chosen phages. In MEGA (v11), the phylogenetic tree was built [62].

### Cytotoxicity of phage

The MTT assay was used to assess the impact of phage RM_A1 on the survival of normal human skin fibroblast (HSF) cells; the 3-(4,5-Dimethylthiazol-2-yl)-2,5-diphenyltetrazolium bromide (MTT) assay was employed. HSF cells were cultured in high-glucose Dulbecco’s Modified Eagle’s Medium (DMEM, Biowest, France) with 100 units/mL penicillin, 100 mg/mL streptomycin, and 10% fetal bovine serum added as supplements. 96-well cell culture plates (CELLSTAR, Greiner Bio-One, Portugal) were used for seeding, and the cells were then cultured at 37 °C in a carbon dioxide incubator. Following a 10-fold serial dilution in DMEM media, a pure lysate of RM_A1 in SM buffer was tested on a proliferative monolayer of fibroblasts in the wells at several doses (10^10^,10^9^,10^8^, and 10^7^ PFU/ml). After 24 h, the impact of the phage on the cell growth was assessed. Following incubation, 100 µl of new DMEM with 10% MTT labeling reagent (final concentration 0.5 mg/ml) was added to the utilized DMEM media, and the mixture was then incubated for four hours. The media was then carefully removed, and each well received an equivalent volume of DMSO to dissolve the formazan crystals that had developed over the course of 20 min. A FLUOstar Omega Microplate Reader was then used to measure the absorbance of the wells at 570 nm. The vitality of the phage-treated cells was determined using the OD values and expressed as a percentage of the untreated cells (negative control) using the formula below [63]. Three replicates were used for the cell viability test.

$$\cdot\;\text{Cell viability}\;=\;100-\;\big(1\;-\;\frac{\mathrm{OD}\;\mathrm{phage}\;\mathrm{treated}\;\mathrm{cells}\;-\;\mathrm{OD}\;\mathrm{blank}}{\mathrm{OD}\;\mathrm{untreated}\;\mathrm{cells}\;-\;\mathrm{OD}\;\mathrm{blank}}\big){\%}$$

### Prophylatic and therapeutic effect of *Acinetobacter* phage RM_A1 against *A. baumannii* on HSF cell line

To assess the efficacy of the phages in eliminating the *A. baumannii* in the presence of the cell lines, we investigated two approaches: prophylactic and therapeutic regimens. Initially the therapeutic method was performed within DMEM media, supplemented with 10% fetal bovine serum, 100 I.U./mL penicillin, and 100 µg/mL streptomycin, and the cells were cultivated at 37 °C with 5% CO_2_. After the cells reached confluency, they were trypsinized and cultivated in 96-well plates with a seeding density of 5 × 10^4^ cells/well. They were then incubated for 24 h at 37 °C with 5% CO_2_. In parallel, an overnight culture *of A. baumannii* was made and incubated at 37 °C. Following a 24-hour period, the bacterial culture was centrifuged for one minute at 11,000 rpm, resuspended in PBS, and its optical density (OD) was determined to be 10^6^ CFU/ml. After centrifuging once again, the bacterial culture was resuspended in full RPMI medium without antibiotics. Following the test of cell adhesion on a 96-well plate, the old medium was discarded, washed twice with PBS, and 100 µl of bacterial suspension per well was added to achieve a final concentration of 10^5^ CFU/well. At 37 °C and 5% CO_2_, the cells were incubated for one hour. After the removal of the supernatant from every well except the control group (HSF cells alone), 100 µl of phage was added to each well with a variety of MOI (0.1, 1, 10, and 100). Next, 100 µl of RPMI medium without antibiotics was added to each well. The plate count approach was used to measure the bacterial count and phage titer at zero, two, six, and twenty-four hours. The cells were counted after 24 h by discarding the supernatant, washing twice with PBS, adding 40 µl of trypsin, and then incubating for 15 min. Next, 60 µl of full RPMI medium was added, properly mixed, and cells were counted using a Trypan blue and hemocytometer [64]. The prophylactic method, on the other hand, involved adding the phage to wells containing HSF cells, incubating them for an hour, and then discarding them before adding the *A. baumannii* culture [65]. This experiment was conducted in triplicate.

### Statistical analysis

Three duplicates of each experiment were conducted, and the mean ± standard deviation (SD) was used to express the results. The software GraphPad Prism 10.5.0 was utilized for data analysis and graph generation using one-way ANOVA.

## Results

### Bacterial identification and susceptibility to antibiotics

**Fig. 1:**
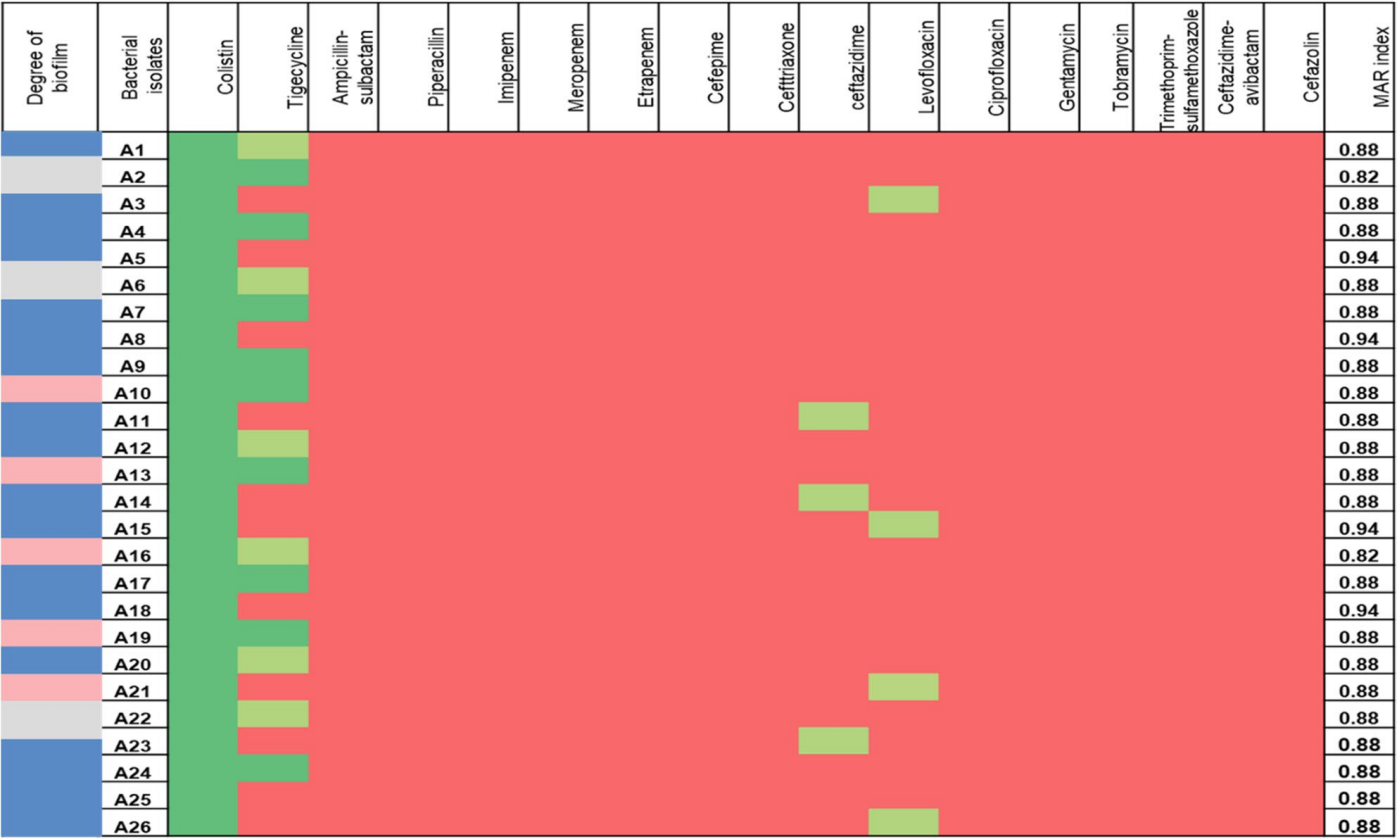
A heatmap of biofilm formation by different strains of *A. baumannii*, with strong formation indicated inblue, moderate inpink, and weak in grey. The antimicrobial profile of the *A. baumannii* isolates against 17 antibiotics from 8 different classes is also displayed (resistant is indicated in red, intermediate in light green, and sensitive in dark green), with the MAR index for all isolates in the left column

Through the automated Vitek 2 system, the twenty-six clinical bacterial isolates were found to be *A. baumannii.* Figure 1 shows the heatmap that displays a complete resistance pattern of 100% to imipenem, meropenem, ertapenem, ceftazidime, cefepime, ampicillin-sulbactam, piperacillin, gentamicin, tobramycin, cefazolin, trimethoprim-sulfamethoxazole, and ciprofloxacin that was displayed by all strains. While piperacillin resistance was approximately 96%, only about 42% of these strains were resistant to tigecycline, with 34% being sensitive and 24% intermediate. About 85% of the strains were resistant to levofloxacin, and approximately 88% were resistant to ceftazidime-avibactam, while all strains were sensitive to colistin. These results confirmed that all strains of *A. baumannii* were carbapenem-resistant, with MAR indices greater than 0.8, indicating an extremely resistant profile. The findings revealed that the strains A5 and A18 had the highest MAR index values.

### Biofilm formation

All of the *A. baumannii* strains formed biofilms with varying degrees. According to the plate reader readings at OD 595, as shown in Fig. 1, the majority of *A. baumannii* strains (about 69%, 18 strains) were able to create strong biofilm, followed by moderate biofilm formation (19%, 5 strains) and weak biofilm formation (12%, 3 strains). The most potent generator of biofilm was the *A. baumannii* A 18 strain.

### Molecular identification of the selected bacterial isolate

The representative isolate, A18, was chosen for additional identification by amplifying and sequencing the 16 S rRNA gene. A single, distinct fragment with a molecular weight of 1166 bp was obtained from the PCR amplificaton after employing 2% agarose gel electrophoresis. The nucleotide sequences were submitted to the NCBI GenBank database in the USA and assigned the accession number PV094173.

### Phage characterization

#### RM_A1 phage morphology

**Fig. 2:**
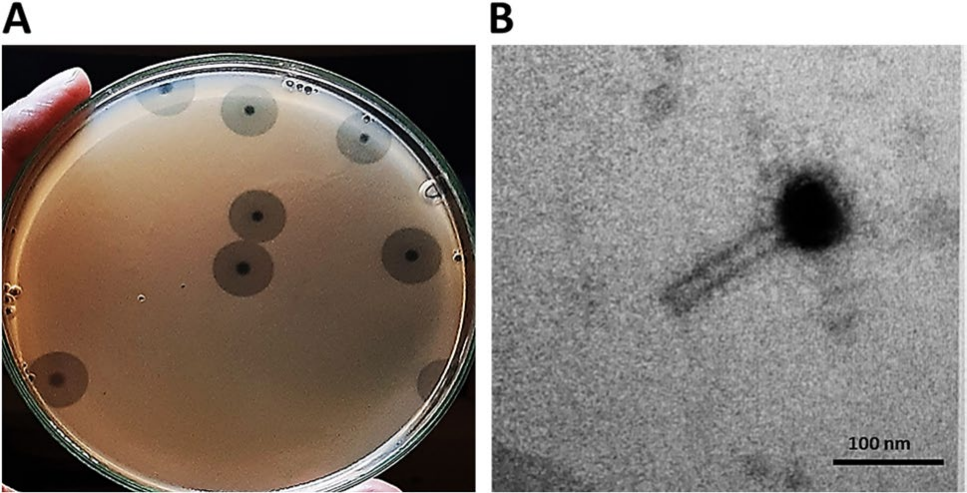
Morphological characteristics of phage RM_A1. **A**: Phage RM_A1 plaque with a halo zone formed in double-layer agar plates on the A18 lawn. **B**: Transmission electron micrograph of RM_A1 showing myoviral morphotype phage after negatively staining with uranyl acetate (the scale bar represents 100 nm)

On TSA plates, the plaque morphology of phage RM_A1 was about approximately 1 mm in diameter, spherical, and transparent, with a halo zone that was about 5.0 mm in diameter surrounding the plaque (Fig. 2A). Based on the morphology, the phage is classified as a myoviral morphotype belonging to Caudovirales according to the standards of ICTV [66]. The phage is composed of a short, contractile tail and an icosahedral head. As recorded in Fig. 2B, the phage had a 90 nm long tail and a 57 nm icosahedral head.

### Host range analysis

Table 1 demonstrates that the RM_A1 phage had inhibition activity against 85% of the studied clinical isolates of *A. baumannii*, indicating a broad host range.

**Table 1:** Host range of RM_A1 phage

Bacterial isolates	Phage host spectrum
A1	+
A2	+
A3	+
A4	-
A5	+
A6	+
A7	-
A8	+
A9	+
A10	+
A11	-
A12	+
A13	+
A14	+
A15	-
A16	+
A17	+
A18	+
A19	+
A20	+
A21	+
A22	+
A23	+
A24	+
A25	+
A26	+

(+) Indicates that the strain is susceptible to the phage, while (-) indicates that the strain is not susceptible

### Relative efficiency of plating (EOP)

**Fig. 3:**
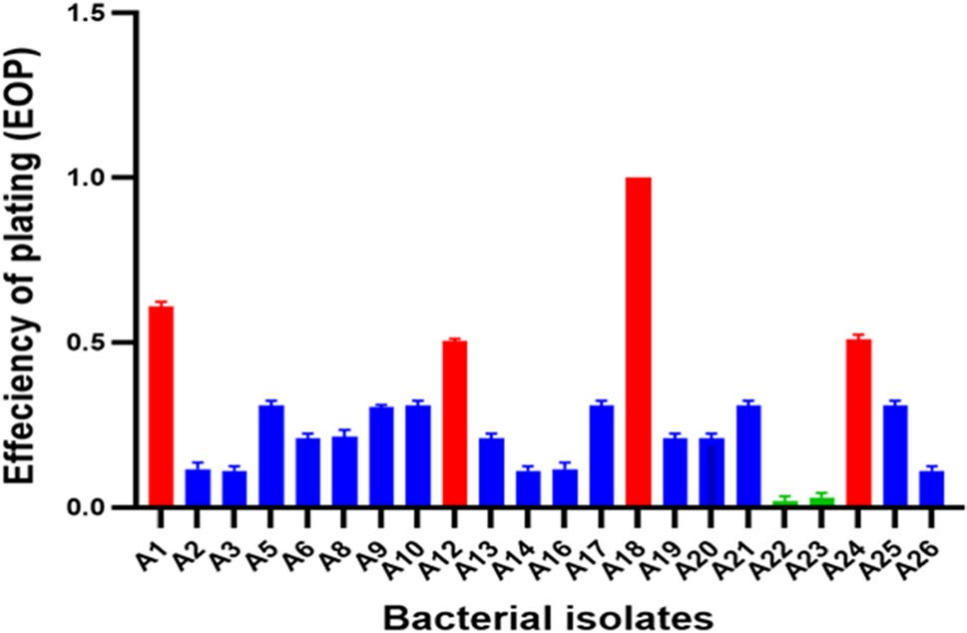
The efficiency of plating (EOP) of RM_A1 phage. A18 is the indicator host with the highest EOP. EOP is categorized as high (red) when EOP is ≥ 0.5, moderate (blue) when 0.5 > EOP ≥ 0.1 and low (green) when 0.1> EOP > 0.001. The mean of triplicate results of each bacterial host was plotted, and error bars represent their standard deviations

Relative EOP was used to further evaluate the phage’s inhibition potential. Because the A18 strain produced the highest phage titer, it was used as the indicator host for calculating the relative EOP. The majority of isolates (16 strains) have medium EOP, with 4 isolates showing a high EOP and only 2 isolates with low EOP as shown in Fig. 3.

### Optimal MOI, adsorption rate, and one-step growth curve

**Fig. 4:**
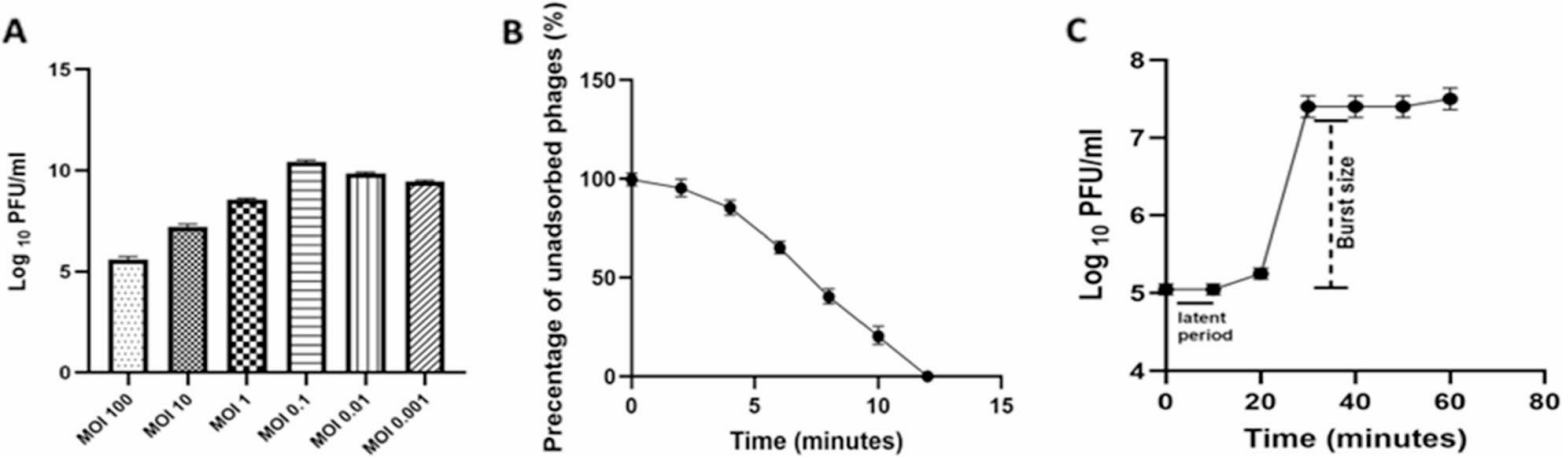
The activity of a phage against the host bacteria. **A**: The phage titer after five hours of incubation with different MOIs. **B**: The adsorption curve demonstrates the adsorption percentage over time as measured by the titer of phages remaining in the supernatant. **C**: The one-step growth curve of the phage. The results were plotted as the mean of triplicates with error bars of ± SD

In the optimal MOI experiment, *A. baumannii* was infected at various MOIs, and the results showed that the MOI of 0.1 was the most effective for achieving the maximum phage titer (10^10^ PFU/mL). Following this, phage with MOIs of 0.01 and MOI 0.001 were more effective than those with MOIs of 1, 10, and 100 (Fig. 4A). The results of the adsorption rate assay, represented in a line graph, displayed the percentage of the unadsorbed phages over a given period. *A. baumannii* fully absorbed the phages in approximately 12 min after exposure, indicating a quick adsorption rate (Fig. 4B). The one-step growth test was conducted at MOI 0.1 to determine the latent time and burst size. The latent duration, according to the curve, was about 20 min. Since the preparation time was also 10 minutes, the entire procedure from adsorption to release took 30 min. Furthermore, the burst size was around 156 phages per single bacterial cell host (Fig. 4C).

### Phage temperature, pH, and UV stability

**Fig. 5:**
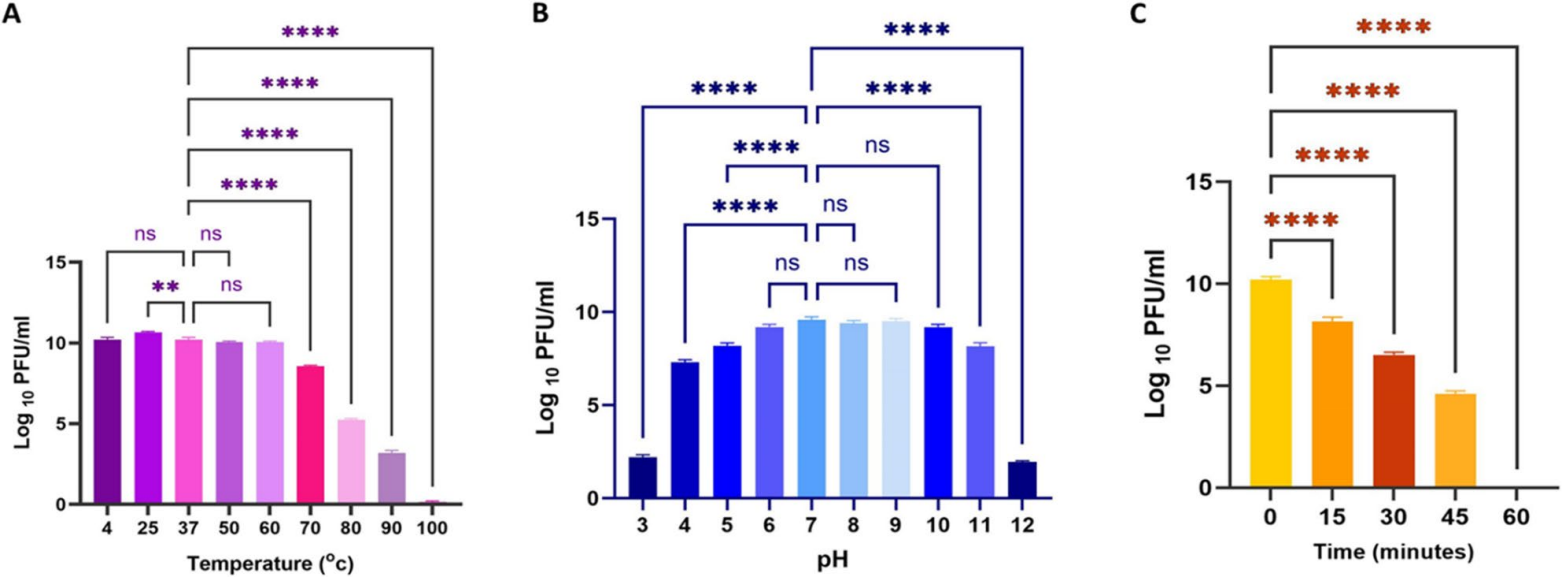
Phage stability at different ranges of temperature (**A**), pH (**B**), and UV exposure **C**. PFU: Plaque-forming unit. Statistically significant differences are marked by asterisks, where **indicates (*P* < 0.05), ****indicates (*P* < 0.0001 and “ns” indicates no significant difference

As illustrated in Fig. 5, the stability of phage RM_A1 was examined for an hour at various pH, UV, and temperature levels. At varying temperatures of 4, 25, 37, 50, and 60 °C, phage RM_A1 titers were constant for 60 min at 10^10^ PFU/mL. However, the titer of the phage dropped to 10^5^ PFU/mL when it was incubated at 80 °C. It then gradually decreased at 90 °C, while it drastically dropped below the detection limit when incubated at 100 °C. These findings suggested that the phage could withstand a range of temperature conditions (Fig. 5A). Phage RM_A1 titer was approximately 10^10^ PFU/mL when maintained at pH range from 6.0 to 11. However, its titers sharply dropped at pH 3, 4, 5, and 12, while at pH 1, 2 and 13, the phage completely lost its activity, as represented in Fig. 5B. As shown in Fig. 5C, the activity of Phage RM_A1 dropped from 10^10^ to about 10^8^ PFU/mL after fifteen minutes of exposure to UV and continued to decrease until it became completely inactive at 60 min.

### AntibacterialActivity of RM_A1 phage

**Fig. 6:**
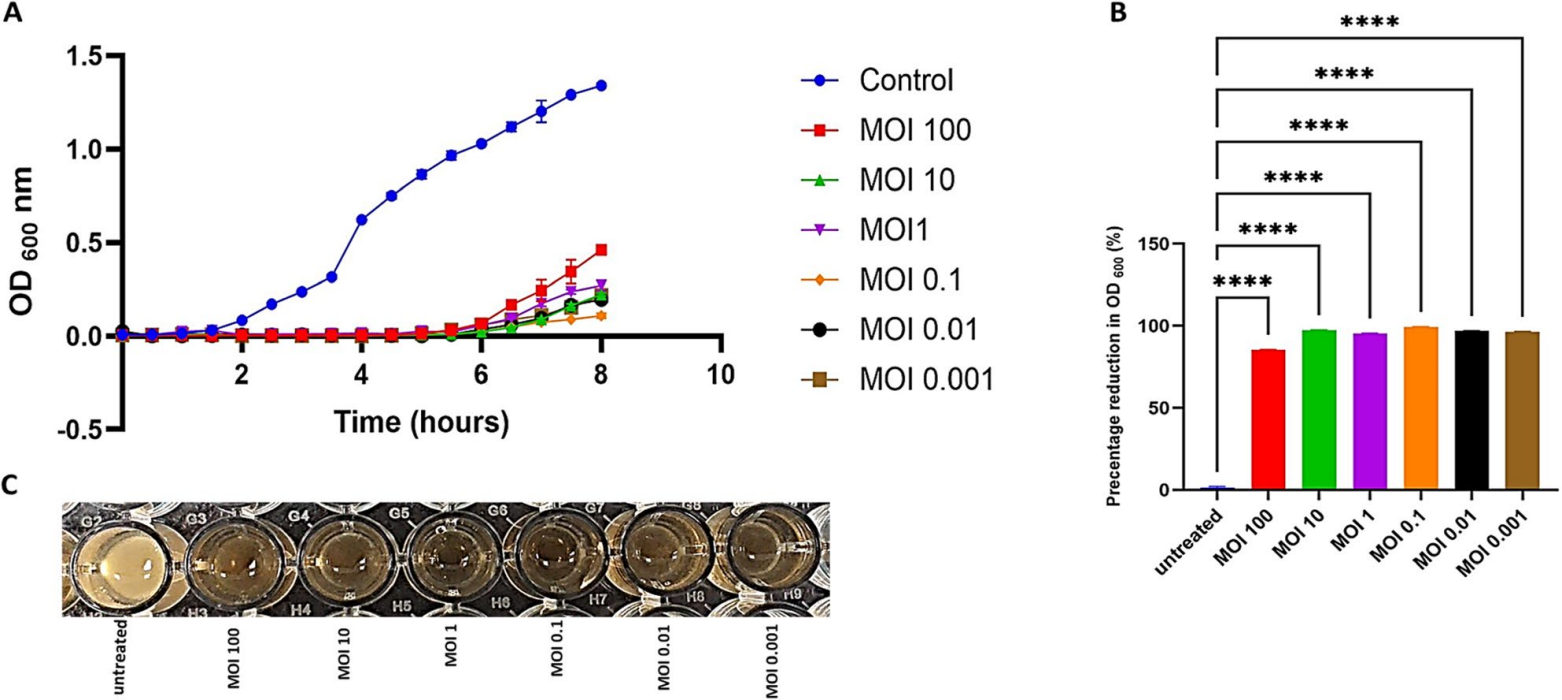
Kinetics of the phage-bacteria interaction RM_A1 against *A. baumannii* at 37 °C. The bacterial growth (OD at 600 nm) of untreated *A. baumannii* (control) and bacteria treated with phage RM_A1 at various MOIs (0.001, 0.01, 0.1, 1, 10, and 100) over an 8-hour period is displayed in (**A**) time-killing curve. **B** The percentage decrease in OD for bacteria treated with phage at various MOIs following 8 h of incubation. **C** The 96-well plate showing different MOIs, that prevent observable bacterial growth compared to the control. The results were plotted as the mean of triplicates with error bars of ± SD. Statistically significant differences are marked by asterisks, where ****indicates (*P* < 0.0001)

Bacterial inhibition was measured by estimating optical density at 600 nm (OD600). When the RM_A1 phage was incubated with its host bacteria at various MOIs (100, 10, 1, 0.1, 0.01, and 0.001) for 8 h, the optical density (OD 600) of the untreated bacteria continued to rise. However, the bacteria treated with various MOIs showed virtually no change in the OD value over the first six hours. After this, the OD of the bacteria treated with various MOIs increased once more, with the OD of the bacteria treated with a high MOI increasing more than that of a low MOI. The bacteria treated with MOI 100 showed the highest increase, while the bacteria treated with MOI 0.1 showed the lowest increase. This demonstrated that the RM_A1 phage efficiently controlled its host’s growth at different MOIs, as shown in Fig. 6A. Following 8 h of incubation, the percentage of reduction in OD was estimated (Fig. 6B), and the results indicated that all MOIs revealed considerably similar outcomes, except the MOI of 100. The best MOI found to inhibit bacterial growth (no observable turbidity) during the incubation period was phage MOI 0.1, as illustrated in Fig. 6C.

### Biofilm inhibition and clearance

**Fig. 7:**
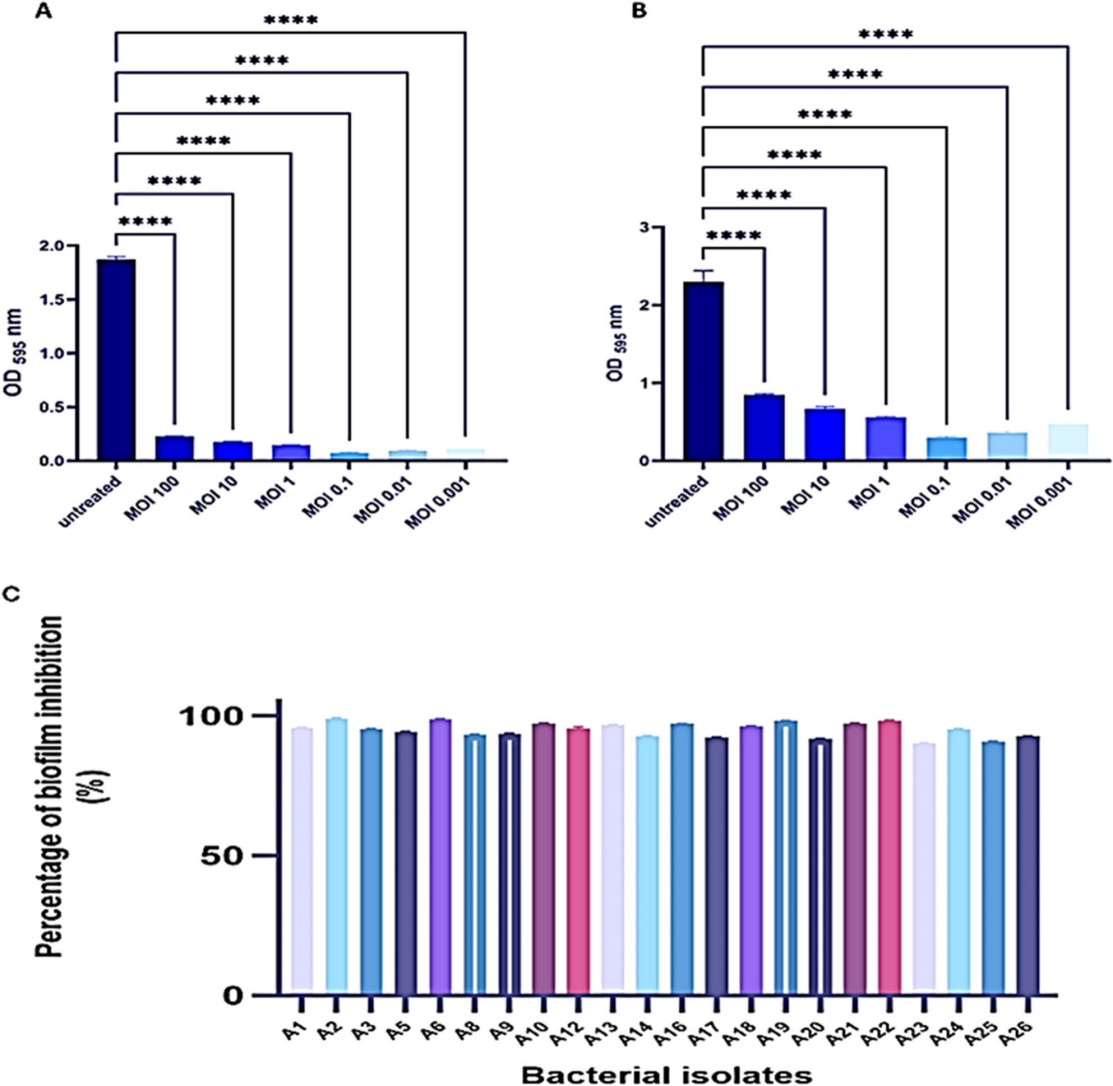
Antibiofilm activity of RM_A1 phage. **A** Phage inhibition of biofilm formation at different MOIs after 24 h in 96-well plates. **B** Clearance of a mature biofilm by RM_A1phage at different MOIs after 24 h in 96-well plates. **C** The RM_A1 phage’s biofilm inhibition percentage against strains of *A. baumannii* examined. Statistically significant differences are marked by asterisks, where ****indicates (*P* < 0.0001)

Phage RM_A1’s antibiofilm activity was experimentally evaluated across a broad variety of MOIs. Both the suppression of biofilm formation and the removal of a biofilm were assessed through the OD measurements. After 24 h of incubation, bacterial biofilm development was considerably lower at all tested MOIs than in the untreated culture (Fig. 7A). The maximum antibiofilm activity was observed at MOI 0.1. The ability of phage RM_A1 to break down mature biofilm was also assessed, indicating its invasive effectiveness. When a 24-hour-old biofilm was subjected to various phage MOIs, the biofilm was successfully disrupted after 24 h for all treated cultures, as their ODs were significantly lower than that of an untreated mature biofilm (Fig. 7B). The lowest OD was observed at MOI 0.1. In conclusion, phage RM_A1 exhibited the best antibiofilm activity at 0.1 MOI, which inhibited the production of biofilms and disrupted the mature biofilm. A calculation of the percentage of inhibition revealed that the highest percentage, 96%, was detected at an MOI of 0.1, followed by 95% at an MOI of 0.01, 94% at an MOI of 0.001, 92% at an MOI of 1, and 90% at an MOI of 10. The lowest percentage, 88%, was detected at an MOI of 100. While calculating the percentage of clearance, the highest percentage, 90%, was detected at an MOI of 0.1, followed by 88% at an MOI of 0.01, 85% at an MOI of 0.001, 80% at an MOI of 1, and 71% at an MOI of 10. The lowest percentage, 68%, was detected at an MOI of 100. The findings showed that whereas the RM_A1 phage had a strong capacity to inhibit the biofilm of all susceptible strains of *A. baumannii*, meropenem (16 µg/ml) had no anti-biofilm action against any of the strains of *A. baumannii* examined. The RM_A1 phage’s biofilm inhibition percentage ranges from 90 to 99% as shown in Fig. 7C.

### Genomic characterization of RM_A1 phage

#### Analysis and annotation of the genome

**Fig. 8:**
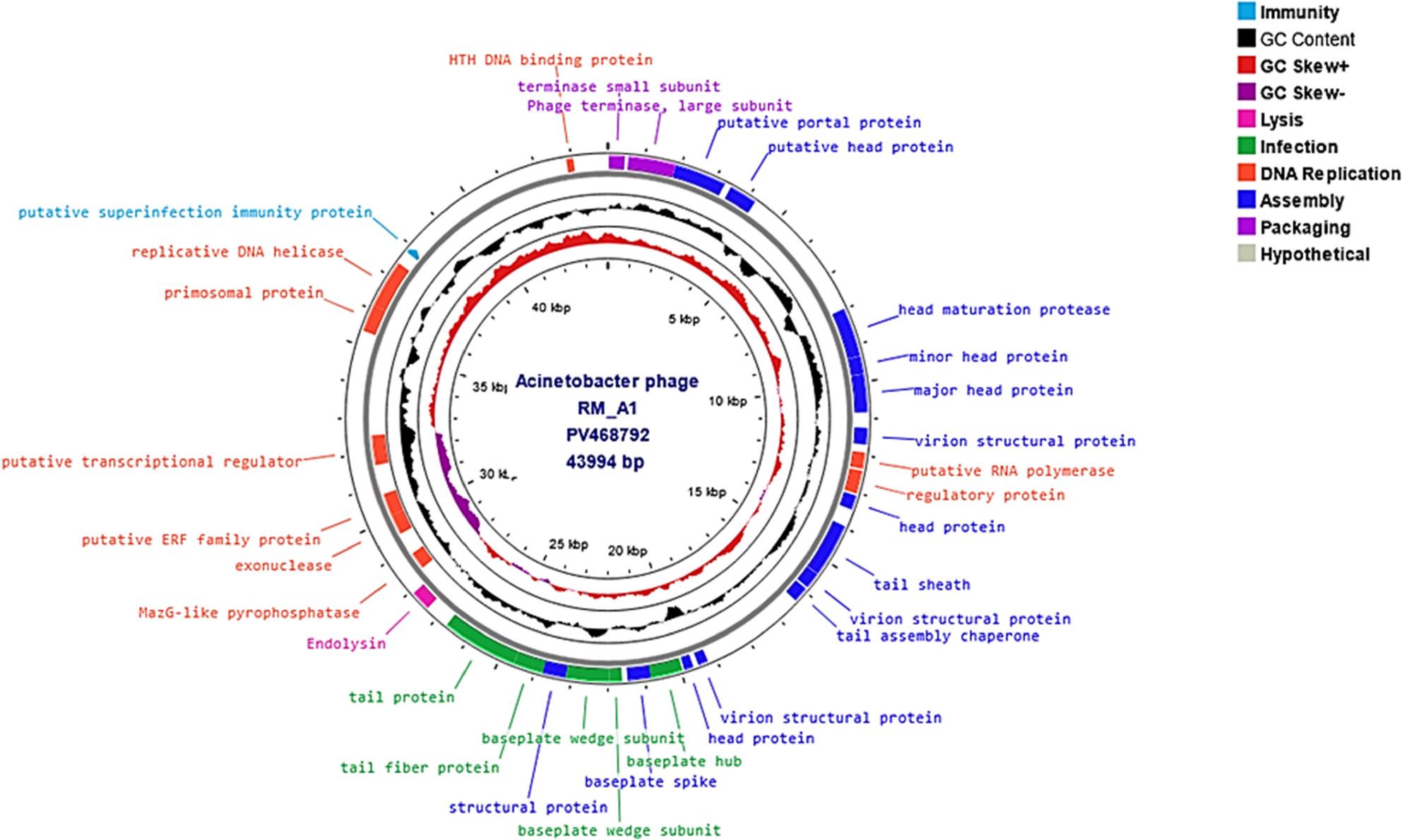
Genomic circular map of phage RM-A1. Colored segments represent coding sequences categorized by predicted function: violet (genome packaging), blue (assembly of virion proteins), pink (lysis), red (replication), turquoise (immunity), green (infection), and grey (hypothetical). The middle circle displays GC content (black). The inner circle shows a GC skew with red for the positive skew and dark violet for the negative skew

A single contig was created from the reads, which had a total GC content of 38% and a double-stranded DNA genome length of 43,994 bp (Accession: PV468792). The whole genome of the RM_A1 phage has 84 predicted open reading frames (ORFs). Thirty-one of these ORFs were assigned to different protein functions, such as DNA genome packaging (terminase small subunit and terminase large subunit), assembly of structure proteins (portal protein, head protein, head maturation protease, major capsid protein, minor capsid protein, virion structural protein, tail sheath, baseplate spike, structural protein and tail assembly chaperone), immunity (superinfection immunity), infection (tail protein, tail fiber protein, baseplate wedge unit, baseplate hub), DNA replication proteins (DNA polymerase, regulatory protein, ERF family protein, Maz G like pyrophosphatase, transcriptional regulation, primosomal, helicase and HTH binding protein) and lysis proteins (endolysin) with no detected tRNA gene. Furthermore, the RM_A1 phage lacked any observable lysogenic phage-related indicators, such as integrase or transposases. Functional genes and their locations on the genome are shown in the genomic map (Fig. 8). According to PHAGESCOPE’s 100% probability prediction, phage RM_A1 has a virulent lifestyle. Furthermore, neither virulence nor antimicrobial resistance genes were detected by PhageLeads, nor were any expected genes associated with a temperate lifestyle.

### Phylogenetic analysis

**Fig. 9:**
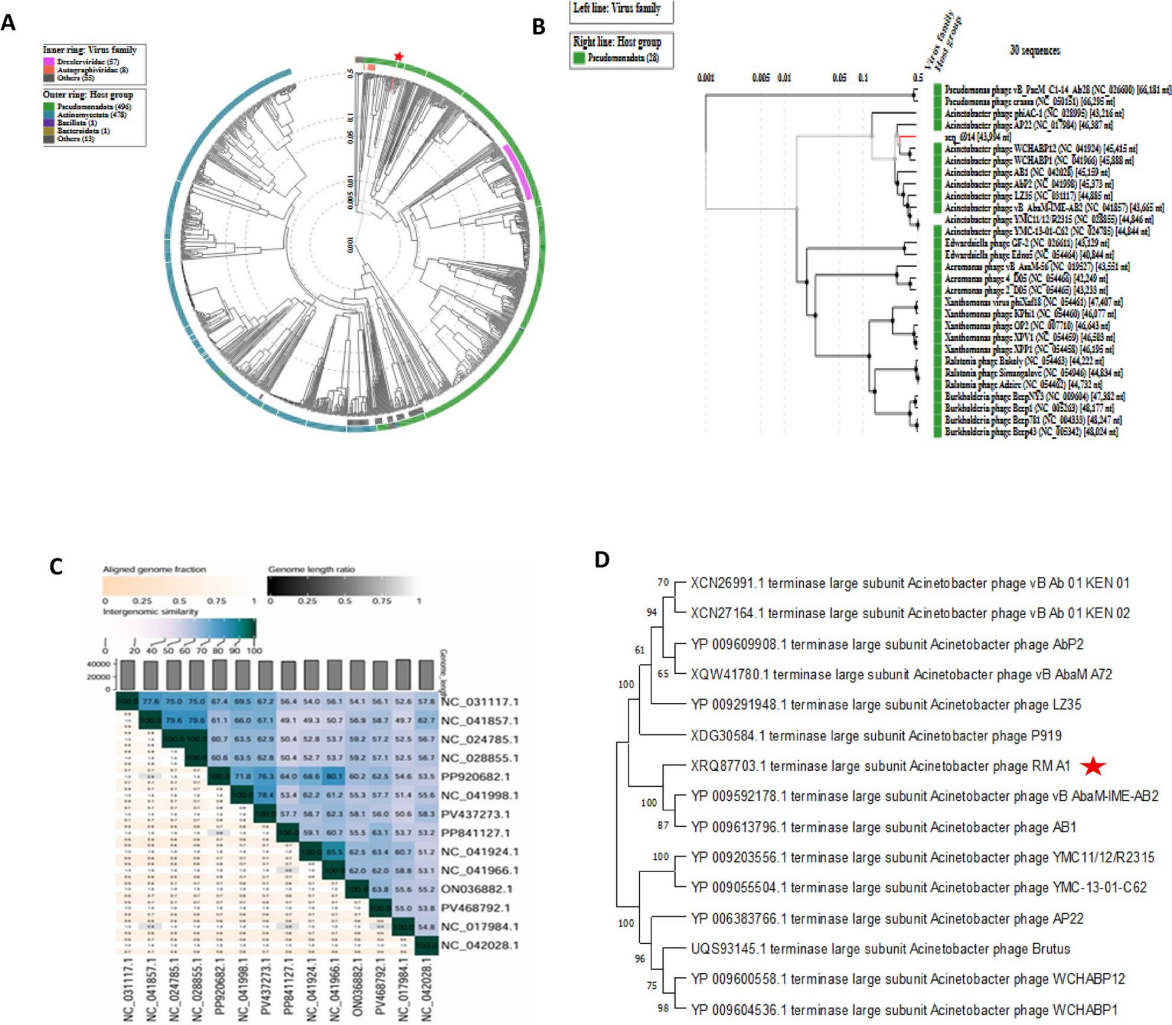
Phylogenetic analysis of phage RM_A1 representing its evolutionary relationships: (**A**) The circular tree comparing RM_A1 to all phages in the ViPtree, with the RM_A1 phage marked by a red star. **B** The rectangular tree highlighting closely related phages with high ViPtree similarity scores. **C** AVIRIDIC heatmap visualizing the intergenomic similarity of RM_A1 to closely related phages, all members of the *Oblenskvirus* genus based on NCBI taxonomy. The heatmap represents clustering at genus and species levels. **D** A maximum likelihood phylogenetic tree comparing the terminase large subunit (TerL protein) of RM_A1, a novel species of *oblenskvirus*, with homologs from other closely related *oblenskvirus* phages and outgrouping phages

Phage RM_A1 clustered among caudovirales with a bacterial host of the class pseudomonada, according to a circular proteome tree created by comparing the phage’s genome to reference phage genomes using ViPTree (Fig. 9A). A more detailed rectangular tree was created using phages with the highest SG scores, and phage RM_A1 was grouped with *oblenskvirus* members (Fig. 9B). The RM_A1 phage was also clustered with other *Oblenskvirus* by VIRDIC’s intergenomic study (Fig. 9C). According to CoreGene 0.5, the terminase large subunit is an ortholog among the genomes of RefSeq phages that belong to the genus *Oblenskvirus*. A phylogenetic tree based on the TerL protein was then created, supporting the idea that phage RM_A1 is a novel species of *oblenskvirus* (Fig. 9D).

### Cell viability assay

**Fig. 10:**
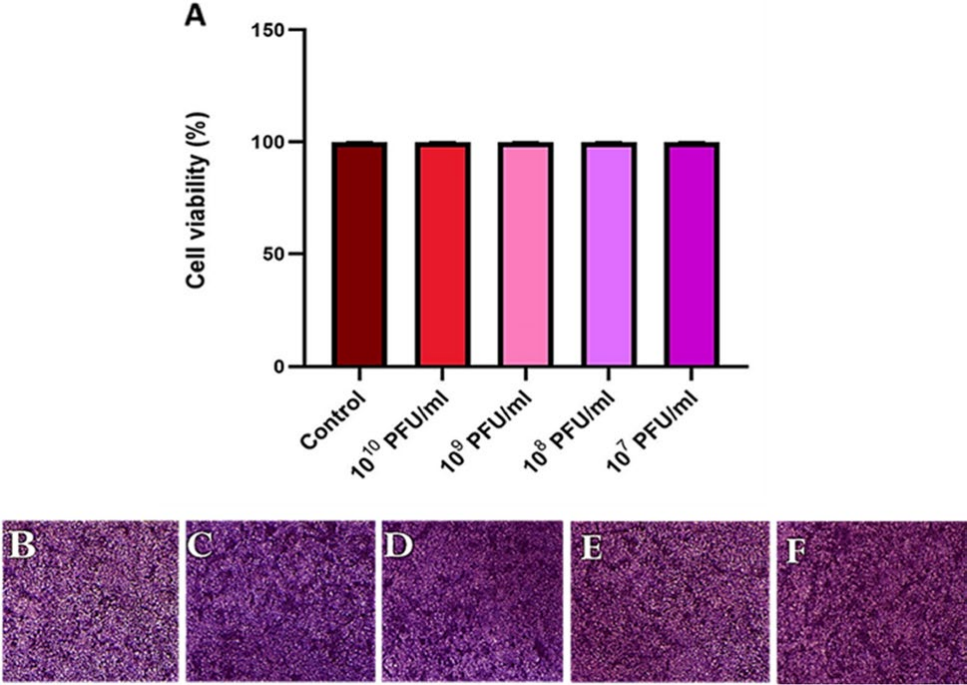
The viability of human cell lines in the presence of RM_A1 phage (**A**) HSF cell viability (%) over a range of phage titers compared to control over 24 h. **B** to **F** photomicrographs of the cell cultures stained with MTT (**B**) the HSF cells without phage (control). **C**, (**D**), (**E**), and (**F**) show HSF cells treated with phage at different titers: 10^10^, 10^9^, 10^8^, and 10^7^ PFU/ml, respectively. The results were plotted as the mean of triplicates with error bars of ± SD.The magnification power used is 10x

As illustrated in Fig. 10, the RM_A1 phage demonstrated safe use with no detectable impacts on the viability of normal cell lines (HSF) when compared to the control, even when applied at a wide range of phage titers (from 10^7^ to10^10^ PFU/ml).

### Prophylatic and therapeutic effect of *Acinetobacter* phage RM_A1 against *A. baumannii* on HSF cell line

**Fig. 11:**
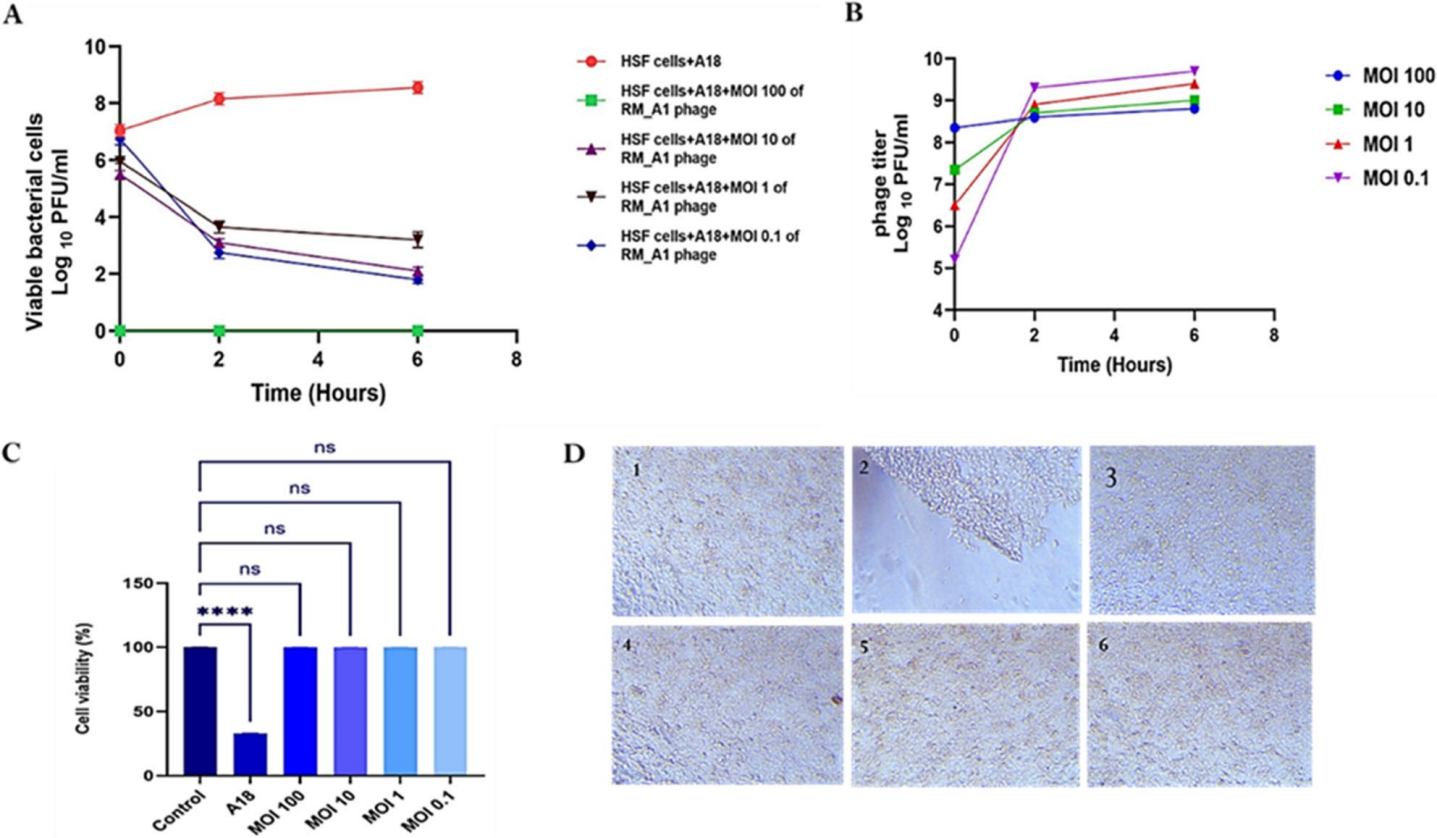
Therapeutic effect of RM_A1 phage against A 18 bacteria in the presence of HSF cells. Viable bacterial cells (**A**), phage titer (**B**), cell viability % (**C**), and HSF cell images (**D**) from 1 to 6): control cells (1), cells with bacteria (2), cells after phage treatment at MOI of 100 (3), cells after phage treatment at MOI of 10 (4), cells after phage treatment at MOI of 1 (5), and cells after phage treatment at MOI of 0.1 (6). The results were plotted as the mean of triplicates with error bars of ± SD. Statistically significant differences are marked by asterisks, where ****indicates *P* < 0.0001 and “ns” indicates no significant difference. The magnification power used is 100 X

**Fig. 12:**
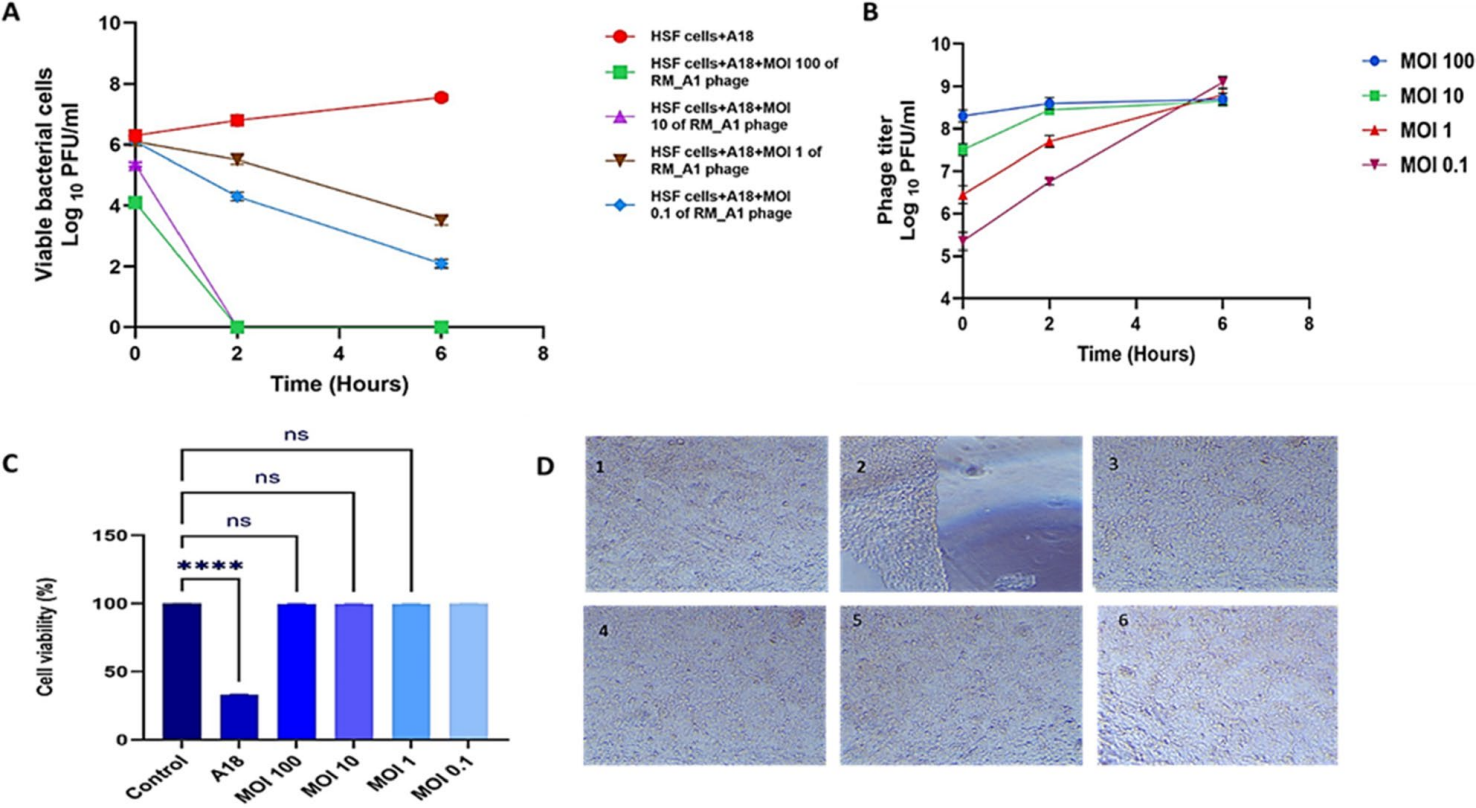
Prophylactic effect of RM_A1 phage against A 18 bacteria in the presence of HSF cells. Viable bacterial cells (**A**), phage titer (**B**), cell viability % (**C**), and HSF cell images (**D**) from 1 to 6): control cells (1), cells with bacteria (2), cells after phage treatment at MOI of 100 (3), cells after phage treatment at MOI of 10 (4), cells after phage treatment at MOI of 1 (5), and cells after phage treatment at MOI of 0.1 (6). The results were plotted as the mean of triplicates with error bars of ± SD. Statistically significant differences are marked by asterisks, where **** indicates *P* < 0.0001 and “ns” indicates no significant difference. The magnification power used is 100 X

The effectiveness of the phage as a preventative and therapeutic agent was assessed using a cell culture method. In both experiments, *A. baumannii* and the phage were co-cultured with HSF cell lines and subjected to different MOIs of the phage. Bacterial survival, phage titer, and HSF cell viability were all evaluated at various intervals (zero, two, and six hours). In the therapeutic experiment (Fig. 11A), our data showed that at time zero, the bacterial count dropped significantly to zero at MOI 100 and was reduced by 2 log_10_ at MOIs of 10 and 1, while it remained unchanged at MOI 0.1. After two and six hours, the number of bacterial cells continuously dropped over time with all MOIs. The bacterial count was still zero at MOI 100. In the prophylactic experiment, the data showed that at time zero, the bacterial count was 10^6^ CFU/ml at all MOIs. After two hours, the number of bacterial cells dropped significantly to zero at MOI 100 and 10, was reduced by one log_10_ at MOI 1, and two log_10_ at MOI 0.1. After six hours the bacterial count was still zero at MOIs 100 and 10, and it had dropped to about 3 log_10_ at MOI 1 and 4 log_10_ at MOI 0.1 compared to the control (Fig. 12A).

In addition to the decrease in CFU, the phage titer increased during the duration of the time points, demonstrating successful phage production (Figs. 11 and 12B). After six hours of incubation, the vitality of HSF cells was unaffected by the presence of bacteria and phages with different MOIs (Figs. 11 and 12C and D); on the other hand, the presence of bacteria alone had a significant impact, reducing the viability of HSF cells to 34% compared to the control.

## Discussion

MDR microorganisms present one of the major challenges to global health. As a result of the rapid increase of bacterial diseases caused by MDR and the decline in the number of newly produced antibiotics, the scientific community is actively looking for alternatives [67]. The use of bacteriophages is one of the potential options. Because of its high host specificity and excellent biosafety, lytic phage has gained acceptance as a natural antibiotic alternative for treating serious bacterial infections [68]. The first phage therapy trial for the treatment of *A. baumannii* infections was approved by the U.S. Food and Drug Administration (FDA) in 2017 [69].

In this study, we examine *A. baumannii*, one of the most critical MDR bacteria. Carbapenem-resistant *A. baumannii* has recently become one of the main pathogens causing ventilator-associated pneumonia, wound infections, and hospital-acquired pneumonia [70].

First, carbapenem-resistant *A. baumannii* isolates were obtained to screen for phage susceptibility. Fortunately, it was not difficult to find MDR *A. baumannii* isolates. The RM_A1 phage was then chosen, characterized, and examined to see if it could lyse carbapenem-resistant *A. baumannii* isolates. The Vitek 2 technique was used to identify the 26 strains of *A. baumannii* that were collected from hospitalized patients in Egypt. Then, they were tested for antibiotic sensitivity using the Vitek 2 AST system, which used 17 different antibiotics from 8 different classes. The results showed significant resistance, which is consistent with multiple studies that estimated the prevalence of carbapenem-resistant isolates from Egyptian hospitals to be between 37.5% and 62.5% [71]. All bacterial isolates showed resistance to 14–16 antimicrobial drugs on average. Every bacterial isolate exhibited a highly resistant phenotype with a MAR index > 0.8. The isolates’ MAR indices indicated that they were obtained from sites with a high burden of resistant bacteria, possibly as a result of overuse of antibiotics. Their antimicrobial profiles demonstrated their resistance to important antimicrobial classes [72]. *A. baumannii* is linked to hospital-acquired illnesses all over the world and quickly becomes resistant to antibiotics through a variety of mechanisms, including multidrug efflux pumps that are effective against a variety of antibiotic classes. One of the main MDR mechanisms in *A. baumannii* is thought to be the inactivation of beta-lactams by beta-lactamases. Acetyltransferases, adenyl transferases, and phosphotransferases are the three groups of enzymes that cause *A. baumannii* resistance to aminoglycoside. Aminoglycosides undergo chemical modification by these enzymes. The target sites for antibiotics are also changed by mutations in DNA gyrase, penicillin-binding proteins (PBPs), and other elements. Certain overexpressed PBPs cause imipenem resistance, and DNA gyrase mutations cause quinolone and tetracycline resistance in *A. baumannii* [73, 74].

Endocarditis, urinary tract infections, septic arthritis, chronic rhinosinusitis, ocular infections, wound infections, and other illnesses linked to medical devices are caused by bacteria that generate biofilms. According to Percival et al. [75], biofilm is also thought to be essential in delaying wound healing and causing persistent wound infections. The detection of biofilm development and the determination of the antibacterial and antibiofilm activity of drugs against biofilm and bacteria within it, respectively, are critical for the prevention of chronic and persistent infections [76]. In this study, the quantitative microtiter plate (MTP) approach was used to detect the formation of biofilm, and the results showed that all strains could produce biofilm to varying degrees. Approximately 69% (18 strains) of the examined strains of *A. baumannii* were able to produce strong biofilm, followed by 19% (5 strains) producing moderate biofilm and 12% (3 strains) producing weak biofilm. The strain that produced the most biofilm was A18, based on the formation of biofilms and antibiotic profile, so it was used for phage isolation and further studies. The 16 S rRNA gene was amplified and sequenced to further identify the representative isolate, A18. The nucleotide sequence was submitted to the NCBI GenBank database in the United States and assigned the accession number PV094173.

The ideal phage for therapy is the one that is safe for human use, highly effective at killing its bacterial host, and entirely lytic. One of the challenges of phage therapy is its limited host range. Phage RM_A1 was isolated and selected for this investigation due to its widest host range, which indicates its inhibition effectiveness, and its ability to target a host with a high level of antibiotic resistance. It exhibited lytic activity against 85% of the clinical isolates of *A. baumannii* under study. The phage lytic capability was further assessed using EOP. Only two isolates had a low EOP, four isolates have a high EOP, and the majority of isolates (16 strains) had a medium EOP. The phage produced clear and homogeneous plaques on a double agar plate with a diameter of approximately 1.0 mm which were surrounded by a diffuse halo that measured approximately 5.0 mm in diameter due to the production of endolysin. Full genome sequencing and TEM morphological investigation both verified that it is one of the myoviral morphotype phages. This is consistent with the findings of Wang et al. [77], who identified the HZY2308 phage, which is similarly categorized under the Caudovirales.

Prioritizing the burst size and latent duration is important when applying phage therapy [78]. Phages with short latent periods and large burst sizes are often more effective in lysing bacteria [79]. In accordance with findings published by Peng et al. [80], which proved that vB_AbaM-IME-AB2 could adsorb its host cells in 9 min with an adsorption rate more than 99% and a short latent period (20 min), phage RM_A1 displayed a short latent period and a short adsorption rate. The phage RM_A1 showed a large burst size of about 156 PFU per infected cell, which is consistent with the results of Wang et al. [77], who reported that the Ab_WF01 phage produces 151 PFU per infected cell. In this work, MOI analysis has demonstrated that the ideal MOI required for phage amplification was 0.1, similar to the findings of Acba_1, Acba_3, and Acba_6, where the maximum phage titer values were found at MOI 0.1 [81]. The higher replication of phage RM_A1 with MOI 0.1 supports the “replication-first” concept since lower initial titers of phage allow for more multiplication in a culture of susceptible hosts.

The RM_A1 phage demonstrated good stability across a broad temperature range (4, 25, 37, 50, 60, and 70 °C). Its titer then steadily declined when incubated at 80 and 90 °C, and reached an undetectable limit when incubated at 100 °C. It was similarly stable across a broad pH range (4–10). Furthermore, it shows flexibility, which makes it practical for application in a range of environmental and physiological conditions. The capsid structure of phages is thought to be responsible for their resistance to extreme physical and chemical conditions, such as heat and pH, according to De Plano et al. [82]. The phage became totally inactive after being exposed to a low pH due to the protein denaturation in the virion. In addition, Langlet et al. [83] demonstrated that the accumulation of viral particles is the cause of the decrease in phage titer at high pH levels. The phage RM_A1 also demonstrated stability in the presence of UV light, which may be due to its linear double-stranded DNA. The phage’s resistance to UV inactivation can be strongly influenced by the type and structure of its genome. For instance, circular single-stranded DNA phages are more sensitive than linear single-stranded RNA phages. The greatest photoreactivation after UV exposure was seen in those with linear double-stranded DNA [84, 85]. These results are in accordance with the findings that Wang et al. [77] reported.

The in vitro antibacterial impact of phage RM_A1 on *A. baumannii* was investigated at different MOIs (100, 10, 1, 0.1, 0.01, and 0.001). Bacterial growth in all MOIs of RM_A1 infected cells decreased to show significant bacterial killing activity and hydrolysis. After six hours of incubation, the bacterial growth increased at all MOI. In contrast to the higher MOI, the low MOI caused a small rise in bacterial growth, which is explained by the fact that the phage is the only antibacterial agent that can multiply over time using a bacterial host [86]. In this study, the low MOI killed the bacteria more effectively than the high MOI after a period because a high MOI phage could recognize and eliminate the bacteria more quickly than one with a low MOI. In all examined MOIs, phage RM_A1 exhibited strong antibacterial activity, confirming its potential as a promising antibacterial agent.

The antibiofilm activity of phage RM_A1 was experimentally studied at a variety of MOIs due to the clinical importance of biofilm development, which increases bacterial resistance and virulence. Evaluations were conducted on the prevention of biofilm formation as well as the breakdown of an established mature biofilm. After 24 h of incubation, bacterial biofilm development was significantly lower at all tested MOIs than in the untreated culture. The greatest antibiofilm activity was demonstrated at an MOI of 0.1. The percentage of inhibition and clearance was calculated, and the results revealed that the highest percentage of inhibition, 96%, was detected at an MOI of 0.1, while the highest percentage of clearance, 90%, was detected at an MOI of 0.1. These results reflect the high ability of phage to eliminate biofilm, either by inhibition or destruction. Both the breakdown of biofilm structures and the suppression of the causal agents are necessary for the treatment of biofilm-associated infections. Bacteriophages use two techniques to demonstrate strong activity against biofilm formations. First, bacteriophages initially penetrate and destroy bacterial biofilms. Second, phage-derived enzymes such as endolysins also have the ability to break down bacterial biofilms [87]. The percentage of biofilm inhibition for each measured MOI concentration showed that phage RM_A1 may specifically target and interfere with the initial stages of biofilm formation. This result is consistent with other studies that have demonstrated the ability of bacterial viruses to interfere with the earliest stages of biofilm formation, such as preventing bacteria from adhering initially or preventing the synthesis of enzymes that act on the biofilm’s extracellular matrix. The percentage of clearance demonstrated that RM_A1 phage could enter biofilm matrices and then degrade them by polysaccharide-degrading enzymes. These results confirm the feasibility of using RM_A1 phage against multidrug-resistant *A. baumannii* associated with biofilm formation.

The whole genome sequencing of phages is essential for understanding their genetic composition, their potential for combating bacteria, and applications in phage therapy and biotechnology. A major issue that interferes with the use of a newly isolated bacteriophage is the risk of its safety and ability to initiate virulence or resistance. Therefore, genome sequencing is necessary to verify its safety by identifying the genes responsible for lysogeny, resistance, or virulence [88].

Different tools were used for the genomic analysis of the phage. The results and the genomic map of phage RM_A1 indicate that its genome contains all essential phage genes for its structure and function, which ensures its safety. In addition, no known genes encoding virulence or antimicrobial resistance genes were detected, making it a promising candidate for therapy. Proteomic analysis was utilized for recognizing the taxonomically distant relationships of phages, particularly at the family level [55]. VIPTree is one such tool that builds a phylogeny based on proteomes. Phage RM_A1 was grouped with RefSeq genome matches at the family level using VIP Tree. Using BLAST analysis, these top matches were also shown to have the closest nucleotide identity. Calculating the intergenomic similarities at the nucleotide level was the main focus of additional study. The phage RM_A1 was grouped with its top BLAST matches in the same genus of *oblenskvirus* by VIRIDIC analysis. Furthermore, a phylogenetic tree was created using signature gene analysis. The RM_A1 phage was shown to cluster within the same genus of *Oblenskvirus* based on the phylogenetic study of the terminase large subunit [89].

At varying doses, the phage showed no noticeable cytotoxic effect against human skin fibroblasts. The lack of harmful effects of phages was also examined and verified by other research, supporting their use in human therapeutic applications [63]. Tu et al. [90] found a higher vitality of phage-treated cells, suggesting that phage exposure may be associated with improved cell attachment.

Animal models are important to evaluate the safety and effectiveness of phages in humans, but they are costly and time-consuming [91]. In addition, the in vitro antimicrobial activity of phages does not directly translate to in vivo activity. Thus, further research is required to learn about in vivo phage activity before advancing to animal models and human trials. The use of cell cultures is one method for testing the efficacy of phages that can provide information about the interaction between bacteria, mammalian cells, and phages in vitro. This method is cost-effective and can yield valuable insights into the behavior of phages in vivo [64].

In this study, we determined the prophylactic and therapeutic efficacy of the RM_A1 phage against carbapenem-resistant *A. baumannii* via the interaction between the RM_A1 phage, *A. baumannii*, and HSF cell lines. The findings revealed that the bacterial count decreased at all MOIs over the incubation period. In the prophylactic experiment, the bacterial count at zero time was 10^6^ CFU/ml at all MOIs. After two hours, the quantity of bacteria decreased by one and two logs at MOI 1 and 0.1 and drastically decreased to zero at MOI 100 and 10. In addition to the CFU decline, the phage titer rose during the duration of the time points, indicating that the phage production was successful. After 6 hours of incubation, the cell viability of the HSF cell line was evaluated, and the findings demonstrated that, in comparison to the control (HSF cells alone), the cell counts were unaffected by any of the chosen MOIs. This demonstrates that the phage could decrease intracellular bacterial invasion and development. These results were in accordance with Essam et al. [92], who reported that the ΦZC3 phage did not harm A549 cells and saved lung cells. After six hours of incubation, the concentration of bacteria was reduced by almost five logs with various MOIs.

These findings demonstrated that the RM_A1 phage can be used as a disinfectant and therapeutic agent for controlling carbapenem-resistant *A. baumannii* because it has been shown to be effective in preventing and reducing the growth of *A. baumannii*.

## Conclusion

In this study, the isolated RM-A1 phage was found to be virulent against carbapenem-resistant *A. baumannii* without any genomic evidence of bacterial resistance or pathogenicity. According to ICTV standards, the RM-A1 phage belonged to *Caudovirales* with a broad host range and excellent stability at different temperatures, pH, and UV values. Furthermore, it possesses strong antibacterial and antibiofilm activity against carbapenem-resistant *A. baumannii* without causing any cytotoxicity to human skin fibroblasts. All these findings make phage RM_A1 a potentially effective alternative treatment approach against carbapenem-resistant *A. baumannii*. Further study is necessary on phage formulations that can be applied topically to wound infections and tested in clinical trials, either alone or in combination with antibiotics or other phages.

## Supplementary Information

Supplementary Material 1.

## Data Availability

The sequence reads of *Acinetobacter baumannii* strain and RM_A1 phage are available in GenBank under accession numbers **PV094173** and **PV468792**.

## Abbreviations

DMEM: Dulbecco’s Modified Eagle Medium
EOP: Efficiency Of Plating
HSF: Human Skin Fibroblast
ICTV: International committee of taxonomy of virus
MAR: Multiple Antibiotic Resistance
MDR: Multidrug Resistant
MOI: Multiplicity of Infection
ORF: Open Reading Frame
PBS: Phosphate Buffer Saline
PCR: Polymerase Chain Reaction
RASTtk: Rapid Annotation using Subsystem Technology Toolkit
TEM: Transmission Electron Microscopy
TerL: Terminase Large Subunit
TSA: Tryptone Soy Agar
TSB: Tryptone Soy Broth
