# Supplementary material for: Prophylactic and therapeutic efficacy of Acinetobacter phage RM_A1 against carbapenem-resistant Acinetobacter baumannii with no cytotoxicity to human skin cells
Source: BMC Microbiol. 2026 Feb 3;26:159. doi: 10.1186/s12866-025-04698-7 (PMC12930648; doi:10.1186/s12866-025-04698-7)
Supplement: Supplementary file 1 — Supplementary Material 1. [file 12866_2025_4698_MOESM1_ESM.docx]

| **Biofilm formation strength** | **OD _595_** | **Bacterial isolates** | **Biofilm formation strength** | **OD _595_** | **Bacterial isolates** |
| --- | --- | --- | --- | --- | --- |
| strong | 1.58 | A14 | strong | 1.70 | A1 |
| strong | 1.04 | A15 | weak | 0.34 | A2 |
| Moderate | 0.63 | A16 | strong | 1.63 | A3 |
| strong | 0.98 | A17 | strong | 1.21 | A4 |
| strong | 1.85 | A18 | strong | 1.27 | A5 |
| moderate | 0.65 | A19 | weak | 0.33 | A6 |
| strong | 1.13 | A20 | strong | 1.13 | A7 |
| moderate | 0.76 | A21 | strong | 1.57 | A8 |
| weak | 0.44 | A22 | strong | 1.56 | A9 |
| strong | 1.01 | A23 | moderate | 0.60 | A10 |
| strong | 1.09 | A24 | strong | 0.96 | A11 |
| strong | 1.22 | A25 | strong | 1.24 | A12 |
| strong | 1.11 | A26 | moderate | 0.66 | A13 |

**Supplementary table 1.** Biofilm formation by different strains of *A. baumannii* which estimated by OD_595_ readings and the strength of biofilm formation which classified into: strong formation, moderate and weak.


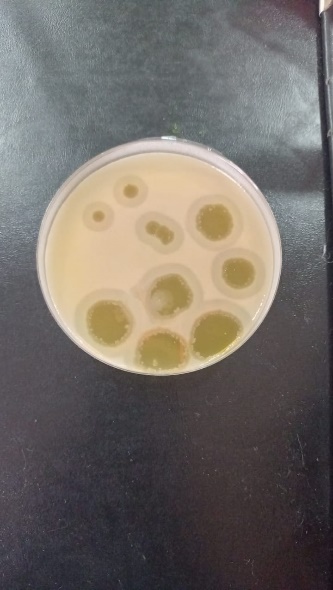

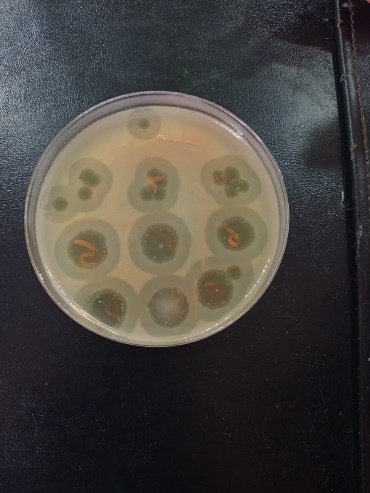

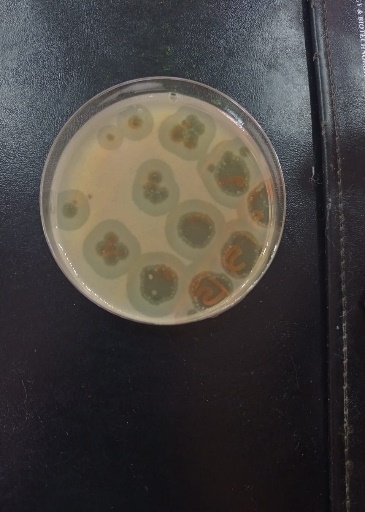


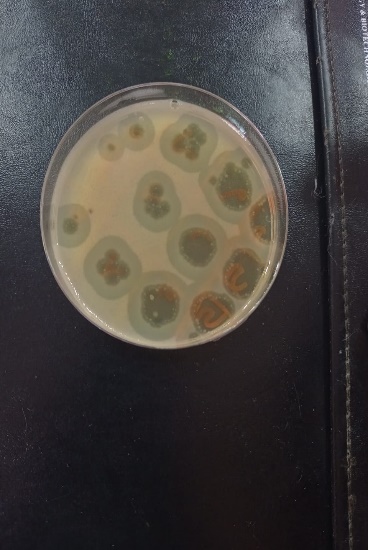

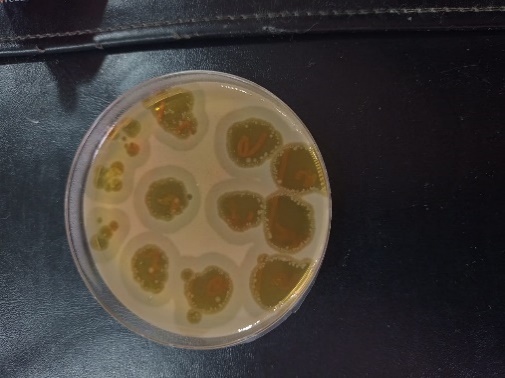


**A6**

**A1**


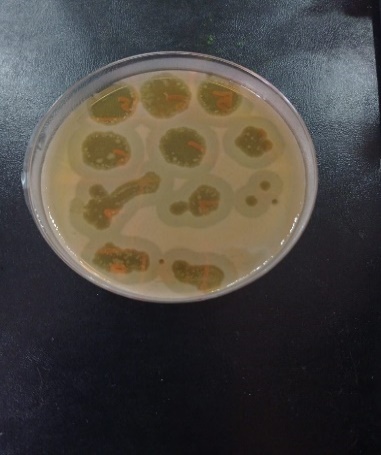

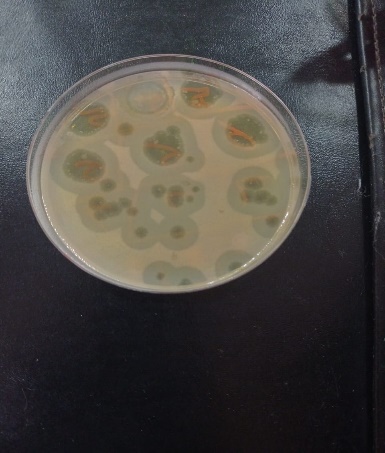

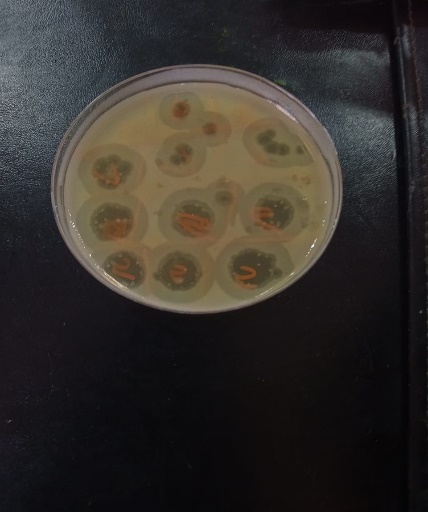


**A5**

**A3**

**A2**


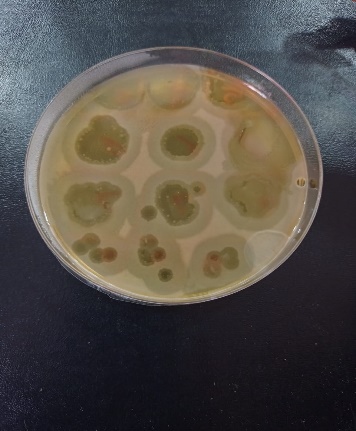

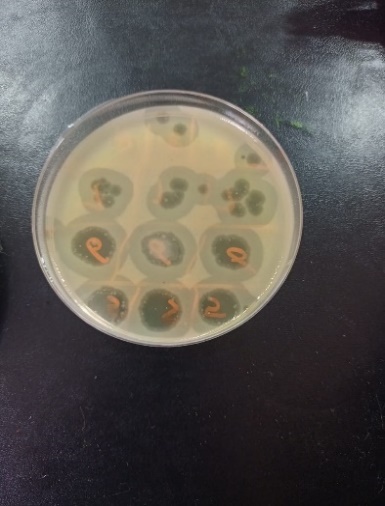

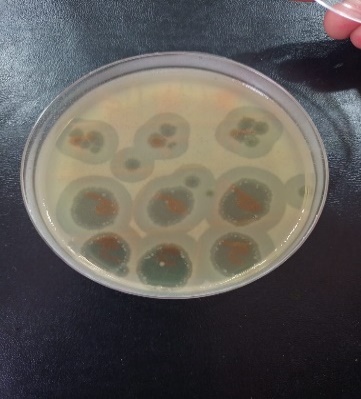

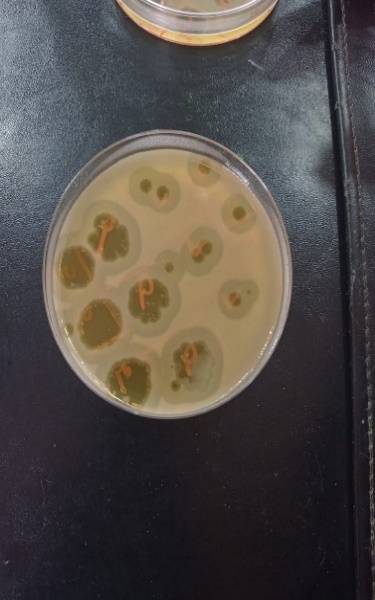

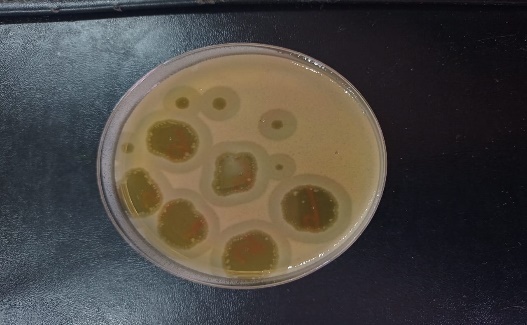


**A13**

**A12**

**A10**

**A9**

**A8**


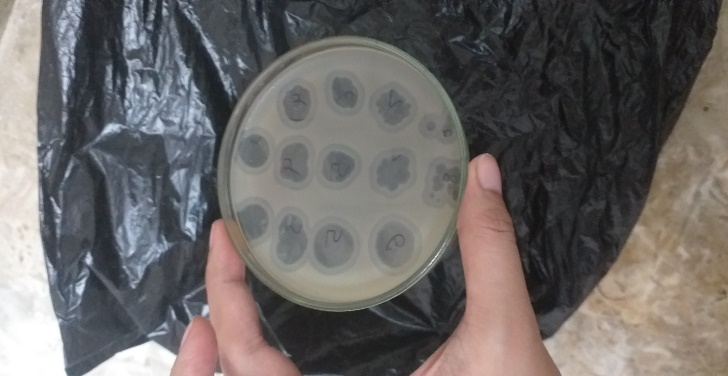

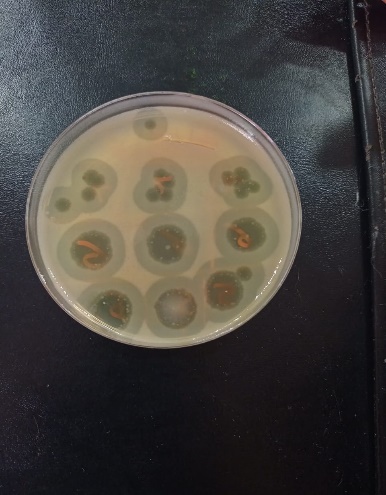

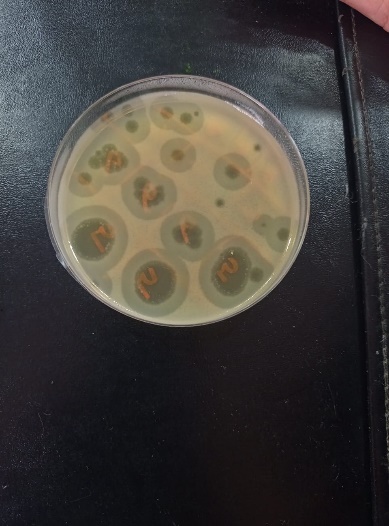

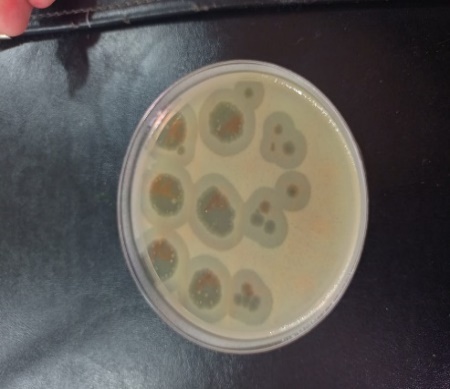

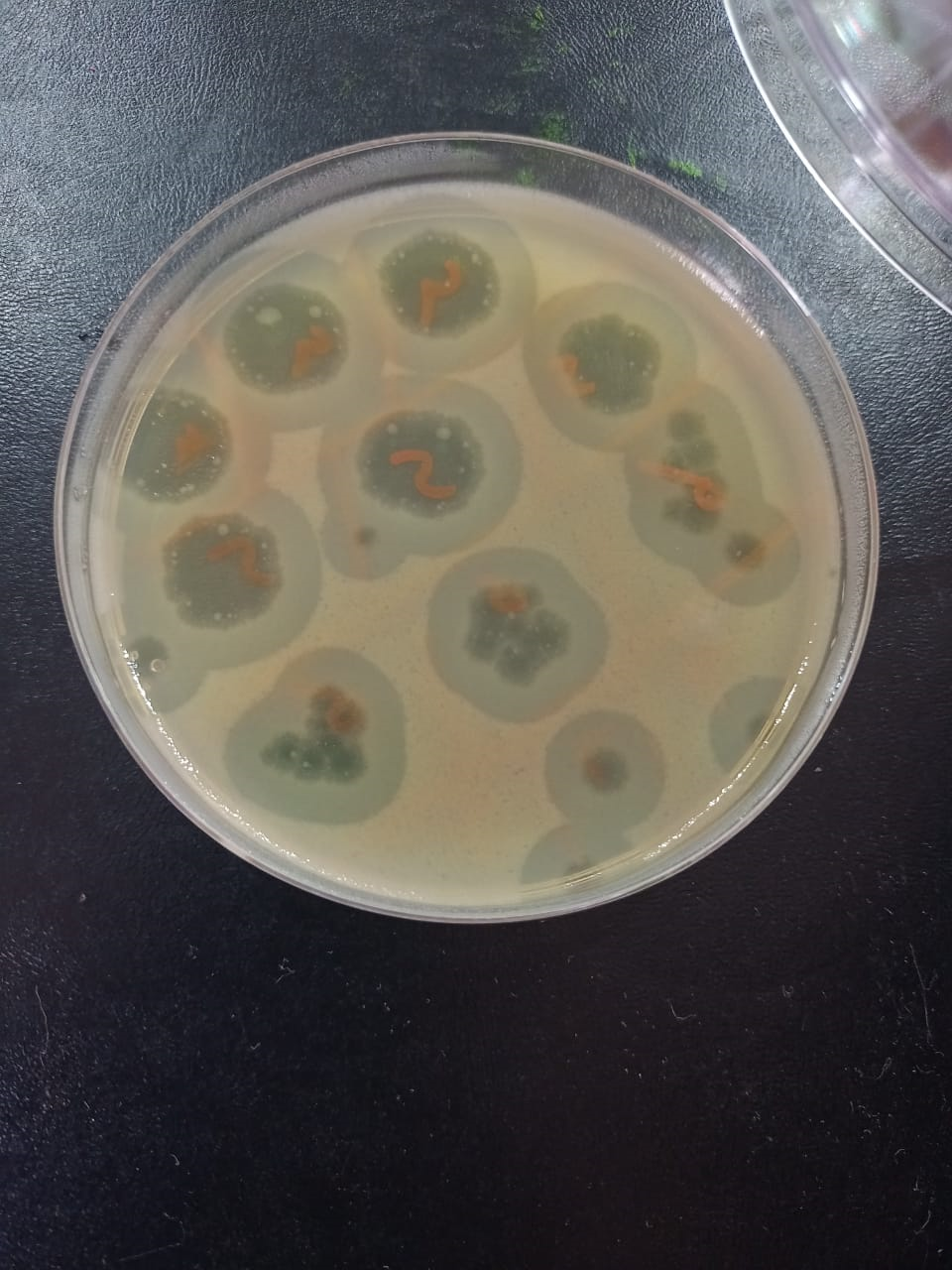


**A19**

**A18**

**A17**

**A16**

**A14**

**
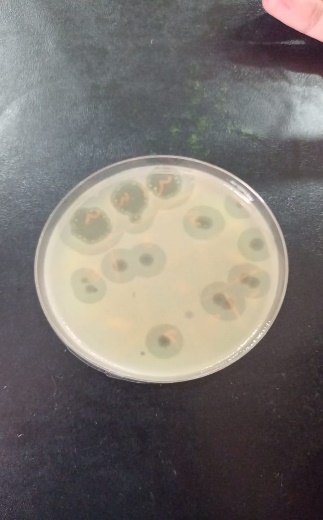

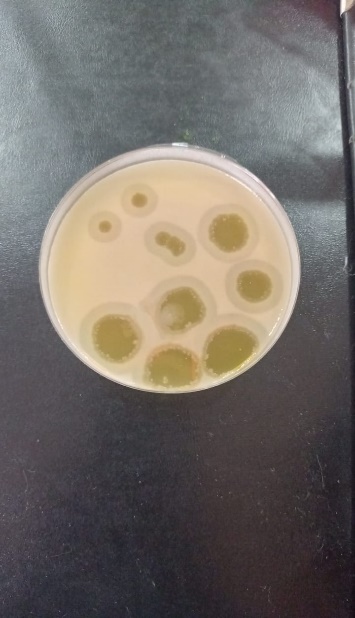

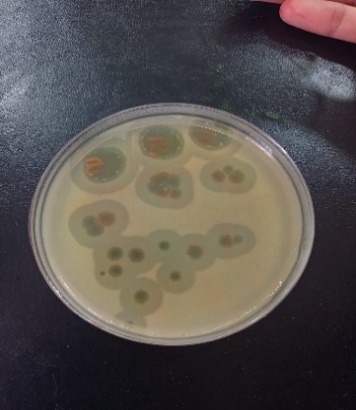

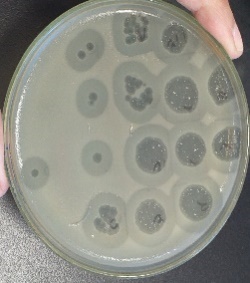

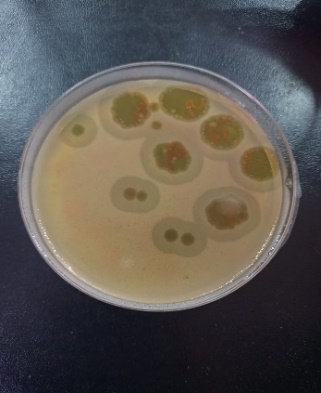
**

**A24**

**A23**

**A22**

**A21**

**A20**

**
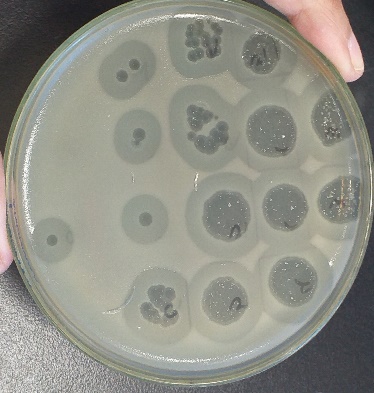

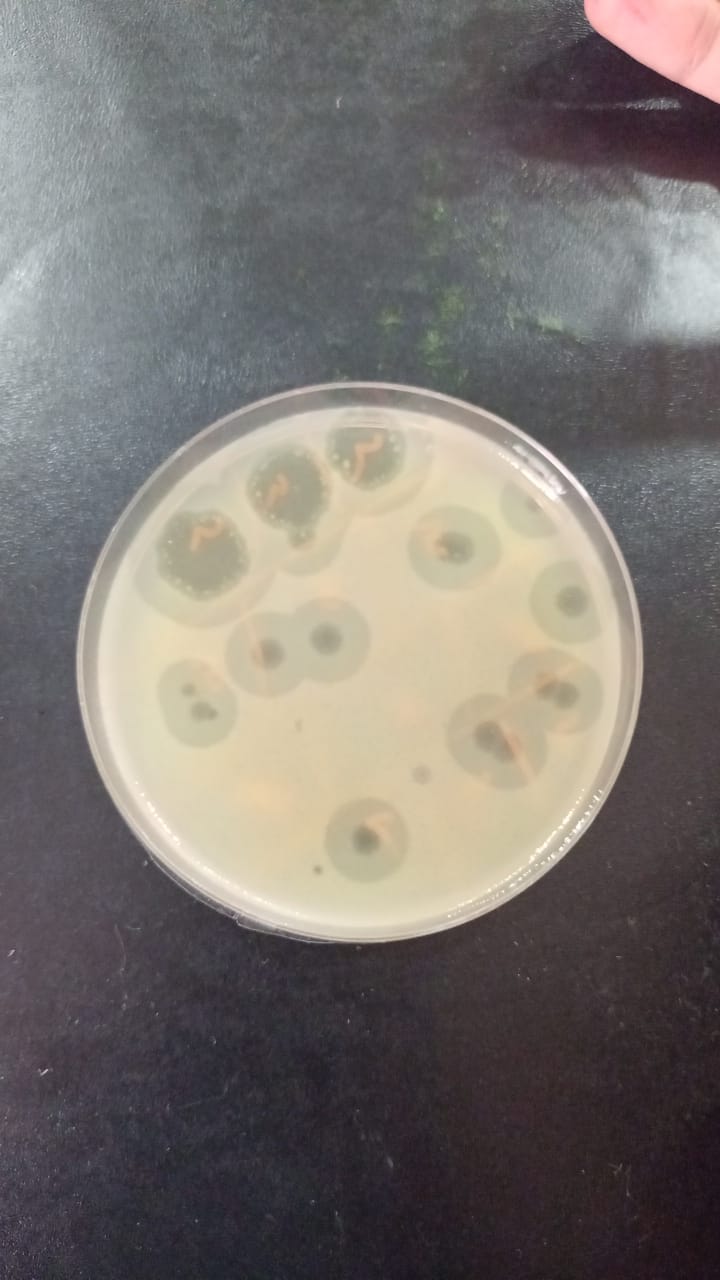
**

**
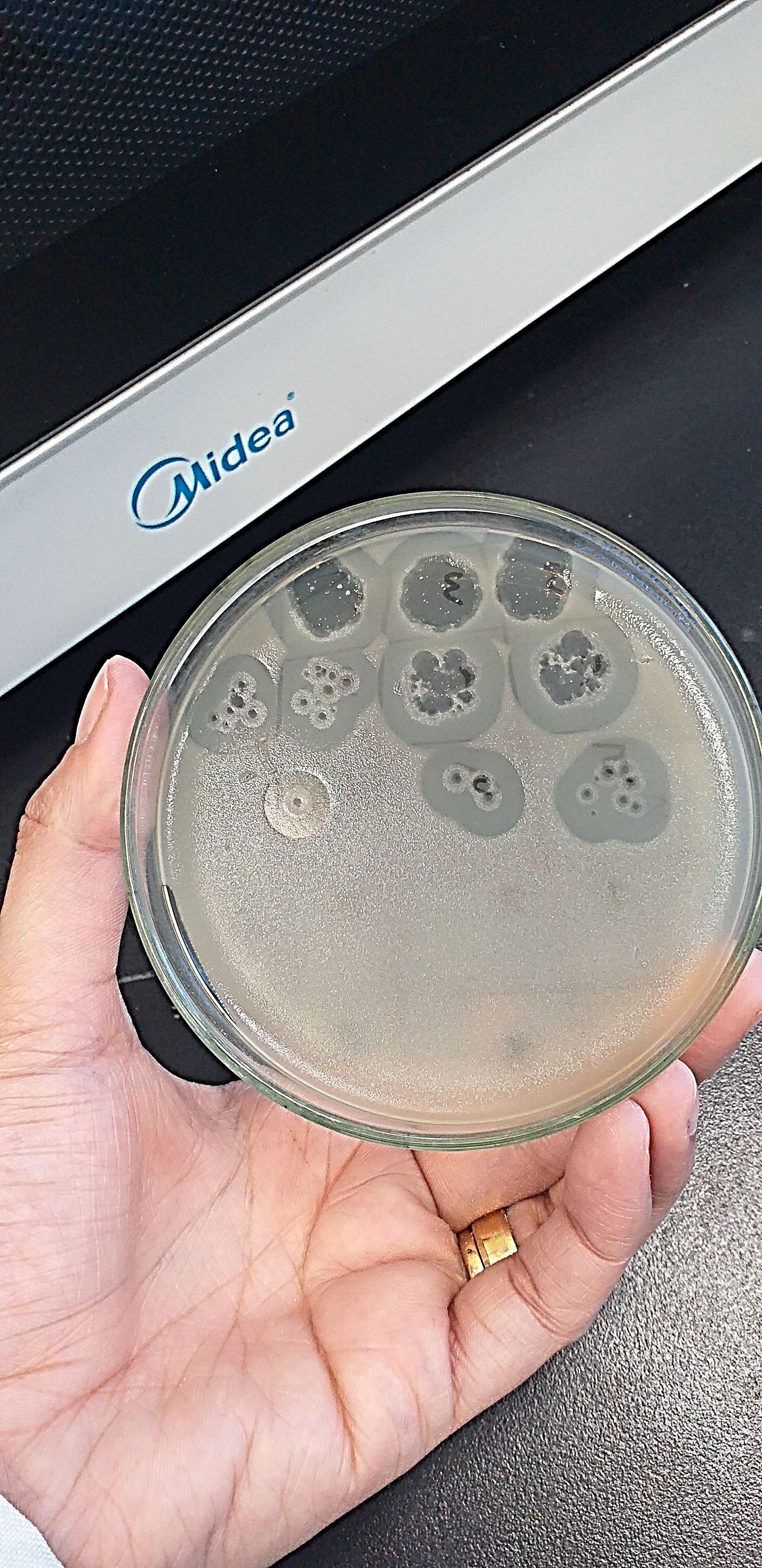

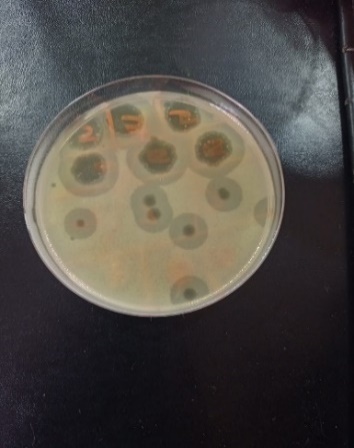
**

**A26**

**A25**

**Supplementary Figure S1.** Spot test demonstrates plaque formation which used for estimating the efficiency of plating of RM_A1 phage for all susceptible strains of *A. baumannii.*
